# Identification of tyrosine-phosphorylated proteins associated with metastasis and functional analysis of FER in human hepatocellular carcinoma cells

**DOI:** 10.1186/1471-2407-9-366

**Published:** 2009-10-16

**Authors:** Haiyu Li, Zhenggang Ren, Xiaonan Kang, Lan Zhang, Xuefei Li, Yan Wang, Tongchun Xue, Yuefang Shen, Yinkun Liu

**Affiliations:** 1 Liver Cancer Institute, Zhongshan Hospital, Fudan University, Shanghai 200032, PR China; 2 Institute of Biomedical Sciences, Fudan University, Shanghai 200032, PR China

## Abstract

**Background-:**

Aberrant activity of tyrosine-phosphorylated proteins is commonly associated with HCC metastasis. Cell signaling events driven by these proteins are implicated in numerous processes that alter cancer cell behavior. Exploring the activities and signaling pathways of these proteins in HCC metastasis may help in identifying new candidate molecules for HCC-targeted therapy.

**Methods-:**

Hep3B (a nonmetastatic HCC cell line) and MHCC97H (a highly metastatic HCC cell line) were used in this study, and the tyrosine-phosphorylated proteins expressed in these cell lines were profiled by a phosphoproteomics technique based on LC-MS/MS. Protein-protein interaction and functional clustering analyses were performed to determine the activities of the identified proteins and the signaling pathways closely related to HCC metastasis.

**Results-:**

In both cell lines, a total of 247 phosphotyrosine (pTyr) proteins containing 281 pTyr sites were identified without any stimulation. The involvement of almost 30% of these in liver or liver cancer has not been reported previously. Biological process clustering analysis indicated that pTyr proteins involved in cell motility, migration, protein autophosphorylation, cell-cell communication, and antiapoptosis functions were overexpressed during metastasis. Pathway clustering analysis revealed that signaling pathways such as those involved in EGFR signaling, cytokine- and chemokine-mediated signal transduction, and the PI3K and JAK-STAT cascades were significantly activated during HCC metastasis. Moreover, noncanonical regulation of the JNK cascade might also provide new targets for HCC metastasis. After comparing the pTyr proteins that were differentially expressed during HCC cell metastasis, we selected FER, a nonreceptor tyrosine kinase, and validated its role in terms of both expression and function. The data confirmed that FER might play a critical role in the invasion and metastasis of HCC.

**Conclusion-:**

The identification of pTyr proteins and signaling pathways associated with HCC metastasis could provide useful information for selecting new molecular intervention targets. Moreover, FER might serve as a novel drug target in future HCC therapy.

## Background

Hepatocellular carcinoma (HCC) is one of the most malignant solid tumors and ranks fourth in terms of mortality and fifth in terms of morbidity among various human cancers worldwide [1]. The short-term prognosis of this condition has dramatically improved due to early diagnosis and surgical resection, transplantation, and local ablation therapy. However, long-term survival is still poor due to frequent intrahepatic metastasis and distant metastasis. Therefore, determining the mechanism of HCC metastasis is a major challenge and impeding the progression of this tumor at an early stage is essential for improving the prognosis of HCC.

Protein tyrosine phosphorylation is an important posttranslational modification that plays a critical role in signal transduction associated with cell proliferation, survival, apoptosis, mobility, adhesion, etc. Tyrosine-phosphorylated proteins (also known as phosphotyrosine proteins, pTyr) include a wide range of signal molecules, such as receptor tyrosine kinases, adapter proteins, and scaffold proteins, which are known to be involved in the cancer metastatic process [2-7]. Aberrant expression and activity of pTyr proteins in the cell signaling pathway have been reported in various human cancers [5,6,8-11]. In recent years, several kinase inhibitors have been introduced for molecular target therapy in clinical oncology. These act by interfering with the activity of specific signaling pathways [9,10,12]. Therefore, exploring and analyzing the expression profiles of pTyr proteins in HCC cells by a high-throughput method would be extremely useful for obtaining insights into the mechanisms of HCC metastasis and recurrence. Such studies would also help in identifying new drug targets for HCC therapy.

In the post-genome era, proteomics techniques based on LC-MS/MS enable us to study the complete range of signal proteins present in a set of cells or tissues under specific conditions [13-15]. In this study, pTyr proteins were identified, and a comparative study was performed between a nonmetastatic HCC cell line (Hep3B) and an HCC cell line with high metastatic potential (MHCC97H). Several biological processes and signaling pathways were found to be strongly correlated with HCC metastasis. Among the differentially expressed pTyr proteins, FER, a nonreceptor tyrosine kinase, appears to be an important protein involved in HCC metastasis.

## Methods

### Cell culture

The Hep3B cell line (nonmetastatic human HCC cells) was obtained from Cornell University, USA, and grown in MEM medium supplemented with 10% fetal bovine serum (PAA) in a 5.0% CO_2 _incubator at 37°C. The MHCC97H cell line (highly metastatic human HCC cells) was established in the Liver Cancer Institute, Zhongshan Hospital, Fudan University [16]. These cells were sustained in DMEM medium supplemented with 10% fetal bovine serum (PAA) in a 5.0% CO_2 _incubator at 37°C. The two cell lines have an integrated HBV DNA genome and exhibit positive alpha-fetoprotein (AFP) expression. These cell lines are commonly used as model cell lines in HCC studies [17-20].

### Immunoprecipitation of pTyr proteins

Cells that had grown to approximately 90% confluency were subcultured. The subcultured cells were grown to approximately 80% confluency and starved in serum-free medium for 12 h to reduce the phosphorylation background resulting from the culture conditions. The cells were placed in a 10-cm dish, washed twice with chilled PBS, and lysed by incubation in 1 ml modified RIPA lysis buffer (150 mM NaCl, 50 mM Tris-HCl (pH 7.4), 1% NP-40, 0.25% sodium deoxycholate, and 1 mM EDTA supplemented with 1 mM sodium orthovanadate, 10 mM sodium fluoride, 10 mM glycerophosphate, and 5 mM sodium pyrophosphate) for 30 min on ice. The cell lysate was cleared by centrifugation at 15,000 rpm for 30 min at 4°C, and the protein concentration was determined by the DC Protein Assay kit (Bio-Rad).

Anti-pTyr specific immuoprecipitation was performed with two different anti-pTyr antibodies. The phosphotyrosine monoclonal antibody pTyr-100 (Cell Signaling Technology) and/or 4G10 (Upstate) was noncovalently conjugated to Protein A-agarose (Sigma) at a concentration of 1 mg/ml beads by overnight incubation at 4°C with gentle shaking. After coupling, the antibody resin was washed twice with PBS and then three times with modified RIPA buffer (5 bead volumes of buffer for each wash). To confirm that efficient coupling had been achieved, an aliquot of the antibody resin was boiled in SDS-PAGE sample buffer for 5 min, and the yield of the released antibody was determined by running it along side a purified antibody standard on SDS-PAGE. The gels were stained with Coomassie blue. The immobilized antibody (400 μl, 400 μg) was added in the form of a 1:1 slurry in modified RIPA buffer to 50 mg cell lysate (1 mg/ml) that had been precleared with 800 μl Protein A-agarose at 4°C for 6 h. The mixture was incubated overnight at 4°C with gentle shaking. The immunocomplex beads were then harvested by centrifugation at 3,000 rpm and 4°C for 5 min and washed three times with 10 bead volumes of modified RIPA buffer. Finally, pTyr-containing proteins were eluted three times with 500 μl of elution buffer (8 M urea, 50 mM NH_4_HCO_3_, and 20 mM ethylamine) for 5 min each at 96°C, and the eluates were combined.

### Desalting and tryptic digestion of pTyr-containing proteins

The pTyr-containing proteins were reduced and alkylated by the ProteoPrep™ Reduction and Alkylation Kit (Sigma) according to the manufacturer's instructions. Briefly, the protein sample was reduced by incubation with 5 mM ributylphosphine (TBP) at room temperature for 30 min. The protein solution was then alkylated by adding 15 mM iodoacetamide and incubating in the dark for 1 h at room temperature. The excess iodoacetamide in the reaction mixture was quenched by additional incubation with 5 mM TBP for 15 min at room temperature. Subsequently, the protein mixture was desalted on a PD-10 desalting column, as recommended by the manufacturer (GE Healthcare), and the buffer was exchanged with a 50 mM NH_4_HCO_3 _solution. Trypsin (modified sequencing grade; Promega, Madison, WI) digestion was performed at a 1:50 trypsin-to-protein ratio (w/w) for 16 h at 37°C. The tryptic peptides were then dried in a Speed Vac.

### Immobilized metal affinity chromatography (IMAC)

Phosphopeptides from trypic digestion were further enriched with the Magnetic Phosphopeptide Enrichment Kit (Clontech), according to the manufacturer's instructions but with a slight modification. First, the tryptic peptides were reconstituted in an appropriate volume of the binding/washing buffer and incubated with the washed and equilibrated beads for 30 min at room temperature by gentle shaking. The supernatant was then removed from the beads by a magnetic separator. The beads containing the phosphopeptides were washed three times with the wash buffer. Finally, the phosphopeptides were eluted three times using a total of 100 μl of elution buffer. The eluates were then filtered through an 0.22-μm filter and dried in a Speed Vac.

### Nanoflow LC-MS/MS protein identification and database searches

The pTyr peptides enriched by IMAC were analyzed using an LTQ mass spectrometer (Thermo Electron Corp., San Jose, CA) equipped with a nanoelectrospray ion source. The *m*/*z *ratios of the peptides and their fragmented ions were recorded by the mass spectrometer. The peak lists for the MS2 and MS3 spectra were generated from the raw data by Bioworks version 3.3 (Thermo Electron) using the following parameters: the mass range was 600-3500, intensity threshold was 1000, and minimum ion count was 10. The generated peak lists were searched by the Sequent program (included in Bioworks) against the nonredundant human protein database of the human International Protein Index (IPI) (ipi.HUMAN.3.3.fasta). The MS/MS spectra were searched with a precursor ion mass tolerance of 2 Da and fragment ion mass tolerance of 1 Da. Full tryptic specificity was applied, two missed cleavages were allowed, and static modification was set for the alkylation of Cys with iodoacetamide (+57). To search the MS/MS data, dynamic modifications were set for oxidized Met (+16) and phosphorylated Ser, Thr, and Tyr (+80). Neutral losses of water and ammonia from the b and y ions were considered in the correlation analysis. To identify phosphopeptides on the basis of the MS/MS or MS/MS/MS spectra, the sequences must meet the following filter criteria: cross-correlation value (Xcorr) > 1.5, 2.0, and 2.5 for singly, doubly, and triply charged ions, respectively; uniqueness scores of matches (deltaCn) > 0.1; and peptide mass tolerance of 5 ppm.

### Bioinformatics analysis of pTyr proteins

For functional annotation and protein-protein interaction (PPI) analysis, the identified pTyr proteins were submitted to the Human Protein Reference Database (HPRD). This database contains extensive information on human proteins, including domain architecture, protein functions, PPIs, posttranslational modifications (PTMs), enzyme-substrate relationships, subcellular localization, tissue expression, and disease association of genes [21-23]. This allows the identified pTyr proteins to be classified into subclasses based on their biological processes and molecular functions. The expression of these proteins was also evaluated under normal and liver cancer conditions using the records in the HPRD. Based on the PPI information available in the HPRD, a PPI network was generated using the Cytoscape version 2.6.1 software [24,25]. This is an open-source bioinformatics software platform for visualizing molecular interaction networks and biological pathways. It allows these networks to be integrated with annotations, gene expression profiles, and other state data. Cytoscape contains some web plug-ins for downloading/linking a network from/to several databases such as pathwayCommons, IntAct, and NCBI Entrez Gene. Moreover, this software contains several plug-ins that allow computational analyses and functional enrichment, such as clusterMaker, BubbleRouter, and BinGO. In this study, functional clustering and signaling pathway analyses of proteins were carried out by running the BinGO, pathwayCommons, and BubbleRouter programs in Cytoscape. BinGO is a tool for determining the Gene Ontology (GO) categories that are statistically overrepresented in a set of genes or a subgraph of a biological network [26]. It returns the correct *p*-value after using two different methods to account for multiple testing. In this study, the result was evaluated by the *p*-value from the Hypergeometric test, and the correct *p*-value was calculated using the Benjamini & Hochberg False Discovery Rate (FDR) correction.

### RNAi and transfection

A 21-nt long double-stranded siRNA for FER was designed on the basis of reported data. It was derived from the human FER cDNA (accession no. J03358) 5'-AAA GAA ATT TAT GGC CCT GAG-3' (nt 84-104) [27] and was synthesized by Jikai Biotechnical Company (Shanghai, China). The selected siRNA sequences were submitted for a BLAST search against the human genome sequence to confirm the specificity of the siRNA. An unrelated silencing sequence was selected and synthesized as a negative control.

For siRNA transfection, 3 × 10^5 ^MHCC97H cells were seeded in a 6-well plate 1 day prior to transfection. Transfection was performed with the Lipofectamine 2000 Plus transfection agent (Invitrogen) according to the manufacturer's instructions. First, 200 pmol siRNA was diluted in 250 μl Opti-MEM (Gibco-Invitrogen) and mixed with 40 μl Plus transfection agent. This was left for 20 min at RT. Next, Lipofectamine 2000 was diluted in 250 μl Opti-MEM and left for 5 min at RT. The two solutions were then mixed and left at RT for 20 min. After aspirating the medium, the cells were washed twice with PBS and added to 1.5 ml of new Opti-MEM and transfection mixture. After transfection for 4-6 h, the medium was exchanged with the standard cell medium. The experiments with FER RNAi were initiated 48 h after transfection.

### Cell invasion assay in vitro

The MHCC97H cell invasion assay was performed in a 24-well plate according to a previously described protocol but with slight modifications [28]. Briefly, 100 μl of Matrigel (1 mg/ml) was added to the upper chamber of a 24-well transwell plate, and the plate was incubated at 37°C for at least 4-5 h to induce gelling. The upper chamber that was coated with Matrigel was washed three times using warm serum-free medium, and 200 μl of cell suspension containing 1 × 10^5 ^cells was placed in the chamber. Simultaneously, the lower chamber of the transwell plate was filled with 600 μl of the cell supernatant from 3T3 cells supplemented with 10% FBS. After cell invasion for 30 h at 37°C, the noninvaded cells at the top of the transwell plate were scraped off with a cotton swab. All invaded cells were stained with the Giemsa staining solution and counted under a microscope. The mean value of three independent experiments was used for the *t*-test to calculate the statistical significance.

### TMA construction and immunohistochemical staining

A tissue microarray (TMA) was assembled using 100 HCC cases, consisting of 50 cases each of HCC with or without metastasis studied over a follow-up period of 2~5 years. For each case, 2 core samples of hepatoma tissue were acquired from a donor paraffin block provided by the Pathology Diagnosis Department of Zhongshan Hospital. Serial tissue sections (4 μm thick) were cut from the TMAs for immunohistochemical analysis.

For immunohistochemical staining, the slides were deparaffinized in xylene and rehydrated through a graded series of ethanol concentrations. Intrinsic peroxidase was blocked by using 3% hydrogen peroxide for 15 min. A solution of 5% BSA in PBST (PBS + 0.125% Tween 20) was used to block nonspecific antibody binding, and anti-FER antibodies (Sigma-ATLAS) were used at the concentration recommended by the manufacturer. The slides were kept overnight at 4°C. After washing three times with PBST for 5 min, the slides were incubated with the secondary antibody for 30 min at room temperature. Following three additional washes in TBST, the slides were developed by DAB staining. The sections were scanned at low magnification. The immunostaining score was estimated on a scale of 0 to 3 based on the percentage and intensity of the stained tumor cells, using the criterion given on the ATLAS web http://www.proteinatlas.org. Whether the stain was distributed on the membrane or in the cytoplasm was also recorded and assessed at high magnification. The immunoreactivity was scored semiquantitatively by counting the area and density of stained cells using the Image-Pro Plus 5.0 software. Manual correction was then performed to normalize the score. Statistical analysis was performed with the SPSS13.0 software using the nonparametric Mann-Whitney U test.

## Results

### pTyr protein capture and the expression profiles of Hep3B and MHCC97H cells

**Figure 1:**
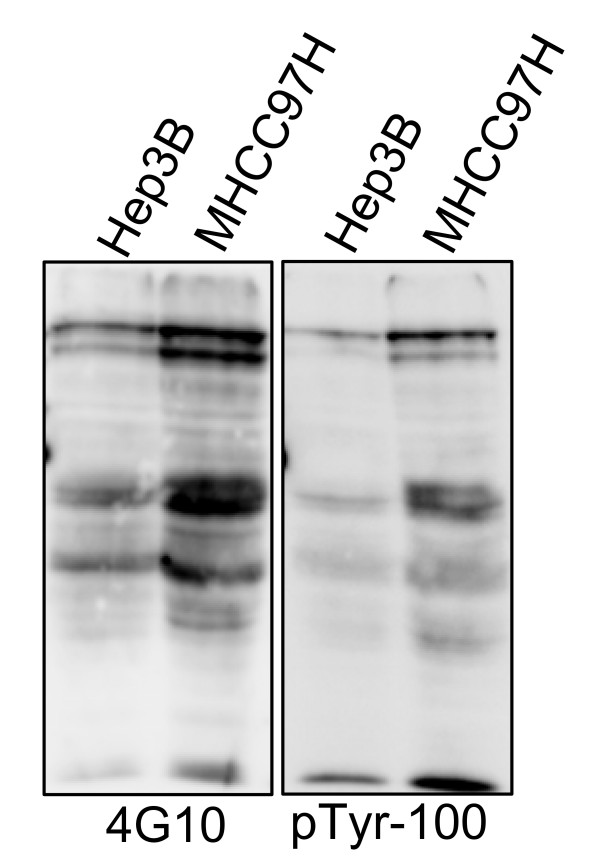
**Comparison of pTyr proteins profile detected using pTyr-100 and 4G10 antibodies in two HCC cell lines with differently metastatic potential**. Protein extracts from two HCC cell lines were separated by SDS-PAGE (20 ug protein per lane) and analyzed by Western blot with anti-pTyr antibodies, 4G10 and pTyr100.

To optimize the enrichment of pTyr proteins, two different antibodies against pTyr proteins were tested for their ability to immunoprecipitate pTyr-containing proteins from the samples. The results showed that 4G10 and pTyr-100 had different protein immunoblotting profiles (Figure 1). The data illustrate the similarities and differences in the pTyr proteins recognized by both antibodies. They also support the use of multiple antibodies to improve the detection of pTyr proteins. In subsequent experiments, a combination of two antibodies was chosen, which was expected to increase the types and quantities of captured pTyr proteins. Figure 1 show that the pTyr proteins in the two cell lines differed in terms of distribution and quantities, suggesting that different pTyr proteins may be involved in HCC metastasis depending on their metastatic potentials.

### Enrichment and identification of phosphopeptides

**Figure 2:**
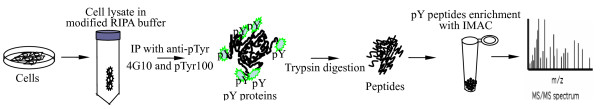
**Scheme of enrichment and identification of pTyr proteins**. Cells were firstly lysed in modified RIPA buffer. pTyr proteins and their interacting partners were then immunoprecipitated by anti-pTyr antibodies. Eluted proteins were further digested into peptides and used to IMAC for second phosphopeptides enrichment. The sample was lastly analyzed by LC-MS/MS.

**Figure 3:**
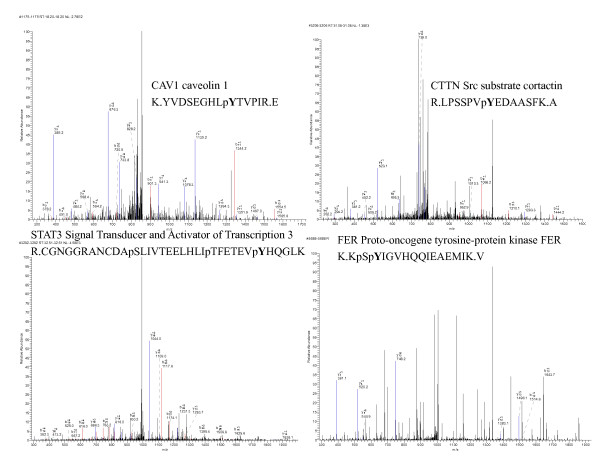
**MS/MS spectra of the pTyr proteins, CAV1, STAT3, CTTN and FER**.

After anti-pTyr immunoprecipitation, tryptic digestion, and IMAC enrichment, the eluted phosphopeptides were analyzed by LC-MS/MS using an LTQ instrument as shown in Figure 2. As described in Materials and Methods, MS/MS spectra were used to search the human IPI 3.3 database with the SEQUENT (version 3.5) software to identify the amino acid sequence and phosphorylation sites. The MS/MS spectra of CAV1, STAT3, CTTN, and FER are shown in Figure 3. In total, 83 pTyr proteins containing 92 pTyr sites were identified in Hep3B cells. The expression of 39 of these has not been reported previously in liver or liver cancer tissues. Similarly, 164 pTyr proteins containing 189 pTyr sites were identified in MHCC97H cells, and the expression of 81 of these was recorded in liver or liver cancer tissues. Among the identified pTyr proteins, 72 pTyr proteins from Hep3B cells and 153 pTyr proteins from MHCC97H cells were differentially expressed. All of the identified pTyr proteins and sites in the two cell lines are listed in Table S1 - S3 in additional file 1. A few phosphoserine/threonine-containing peptides were recovered because their original protein was tyrosine phosphorylated.

### Functional annotation of pTyr proteins

To understand the functions of the pTyr proteins identified in this study, these proteins were submitted to the HPRD database, functionally annotated based on the GO terms, and classified according to their molecular functions (outlined in Tables 1 &2; for detailed information, see Table S4 in the additional file 2). A comparative study on the classification of pTyr proteins in these two cell lines revealed that the differential distribution in biological processes mainly involved protein, nucleic acid, and enzyme/energy metabolism. In terms of the functions at the molecular level, these proteins acted as translation regulators, enzymes, kinases/proteinases, calcium-binding proteins, cytoskeletal-associated proteins, and adhesion molecules. However, there were obvious differences between the Hep3B and MHCC97H cell lines.

**Table 1:** Functional classification of identified pTyr proteins based on biological process in Hep3B and MHCC97H cell

GO-ID: biological process	MHCC97H	Hep3B
Signaling	26%	26%
protein metabolism	11%	4%
nucleic acid metabolism	19%	12%
enzyme/energy metabolism	14%	9%
transport	7%	7%
cell growth and maintenance	5%	6%
novel protein	2%	6%
others	5%	14%
unclassified	11%	16%

**Table 2:** Functional classification of identified pTyr proteins based on molecule function in Hep3B and MHCC97H cell

GO-ID: molecular function	MHCC97H	Hep3B
transcription associated protein	11%	10%
Translation regulatory protein	5%	1%
Receptor	4%	3%
Integral membrane protein	4%	4%
DNA binding protein	1%	2%
RNA binding protein	4%	3%
enzyme	17%	9%
cargo protein	4%	4%
Cytoskeletal associated protein	2%	5%
Calcium binding protein	1%	4%
kinase/proteinase	14%	11%
Ubiquitin proteasome system protein	1%	2%
Adhesion molecule	---	4%
adapter molecule	1%	2%
channel protein	2%	2%
others	7%	6%
Unclassified	8%	12%
unknown	9%	16%

### Protein interaction analysis

**Figure 4:**
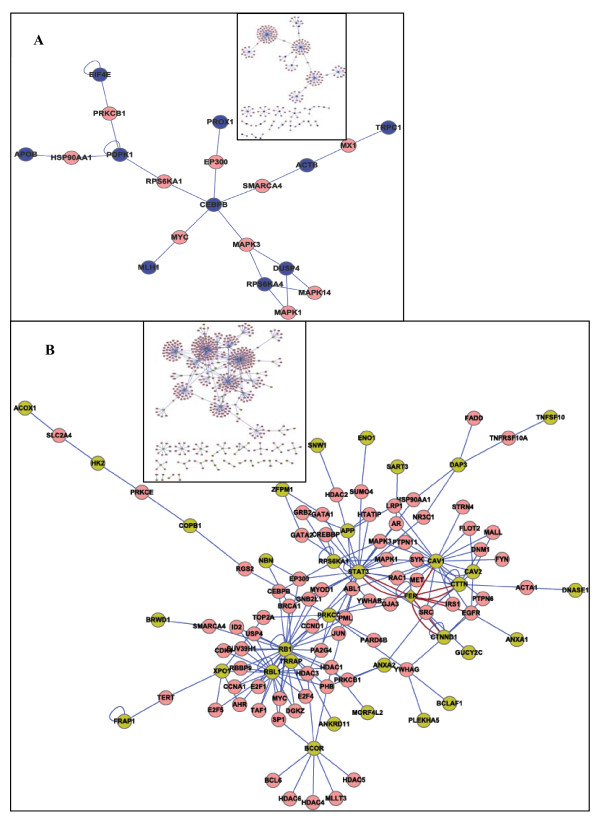
**Simple PPI network for pTyr proteins (root proteins) identified by MS/MS spectrometry with their linker proteins in Hep3B and MHCC97H cell**. Small box represents every complete PPI network for all nodes in two HCC cell lines. A: Hep3B-network B: MHCC97H-network. Pink ellipase node denoted linker proteins, blue and yellow ellipase node represented respectively root proteins in Hep3B and MHCC97H.

A computer-deduced biological PPI network map can represent and indicate the interaction network that exists in a set of proteins. In this, the node molecules represent proteins, while the edges indicate the interactions between proteins. The PPI network can either create PPIs between proteins submitted from MS/MS identification data (root proteins) or it can predict new PPIs between the submitted protein and other proteins from previously published literature. At present, this is believed to be a promising strategy for extrapolating new functions and interactions of biomolecules. To analyze the functional implications of the protein cohorts identified in this study, pTyr proteins that were differentially expressed in Hep3B and MHCC97H cells were subjected to PPI analysis. The 72 proteins that were only expressed in the Hep3B cell line generated a network containing 235 nodes and 224 edges. Similarly, the 153 proteins that were only expressed in the MHCC97H cell line generated a network containing 542 nodes and 596 edges (Figure 4A, 4B upper box). To view the interactions between the root proteins in each network, two simple networks (the Hep3B and MHCC97H networks) that only contained root proteins and linker proteins (interactional proteins found in protein databases, and it bridges two other proteins in the PPI network) were elaborated (Figure 4).

In the two simple networks, linker proteins, including CEBPB, EP300, MAPK1, MAPK3, MYC, PRKCB1, RPS6KA1, and SMARCA4, were shared. These were involved in some important signaling pathways such as the TNF-alpha-NF-kB signaling pathway, androgen-receptor signaling pathway, MAPK cascade, apoptosis pathways, Wnt-signaling pathways, cell cycle-G1 to S control, etc. These pathways play important roles in cell growth, differentiation, cell-cycle control, and apoptosis. The results suggested that the common pTyr proteins involved in these pathways may be associated with HCC pathogenesis.

Moreover, comparison of these two simple networks revealed that FER (a nonreceptor tyrosine kinase) was probably a protein of biological importance in HCC cell metastasis. It existed as only a dual function protein (root and linker-like protein) in these two simple networks. It linked four root proteins--CTNND1, CTTN, STAT3, and CAV1 (see highlighted diamond node and red edge in Figure 4B), in the MHCC97H network; however, it was absent in the Hep3B network. Interestingly, one each of these four proteins played a key role in cytoskeletal regulation, cell adhesion, signal regulation, and transportation. Thus, based on the results, it is reasonable to suppose that FER is an important pTyr protein and was therefore selected for sequential functional validation.

### Functional clustering analysis

To fully predict the coherent function, all node proteins from the PPI networks of the MHCC97H and Hep3B cells were clustered according their GO attributes in biological process and signaling pathways.

**Figure 5:**
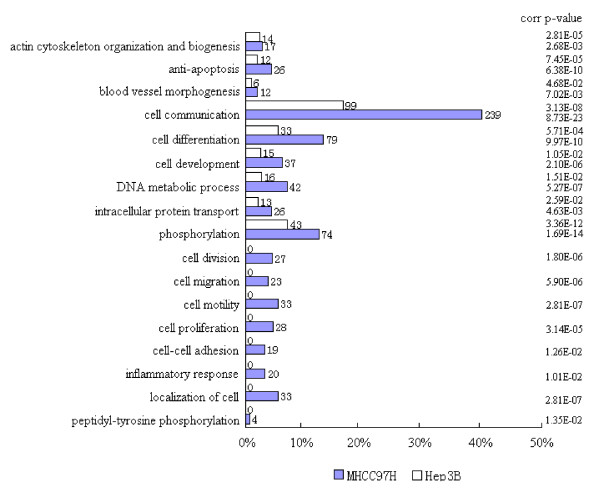
**Histogram of functional cluster analysis based on biological process for all nodes proteins from PPI networks of Hep3B and MHCC97H cell lines**. The numbers of proteins assigned to every functional groups and the significance of the statistical difference are shown.

Biological process clustering analysis showed that in relation to tumorigenesis and metastasis, identified pTyr proteins were abnormally overrepresented in the two PPI networks, especially in the MHCC97H cells (Figure 5). These were mainly involved in cell division, differentiation, development, proliferation, phosphorylation, cell communication, blood vessel morphogenesis, DNA metabolic processes, antiapoptosis functions, intracellular protein transport, cell migration, cell adhesion, and cellular localization. More importantly, biological processes related to cancer metastasis, including cell motility, migration, antiapoptosis functions, cell localization, and cell communication, differed significantly between the two HCC cell lines (*p *< 1.00E-05).

**Figure 6:**
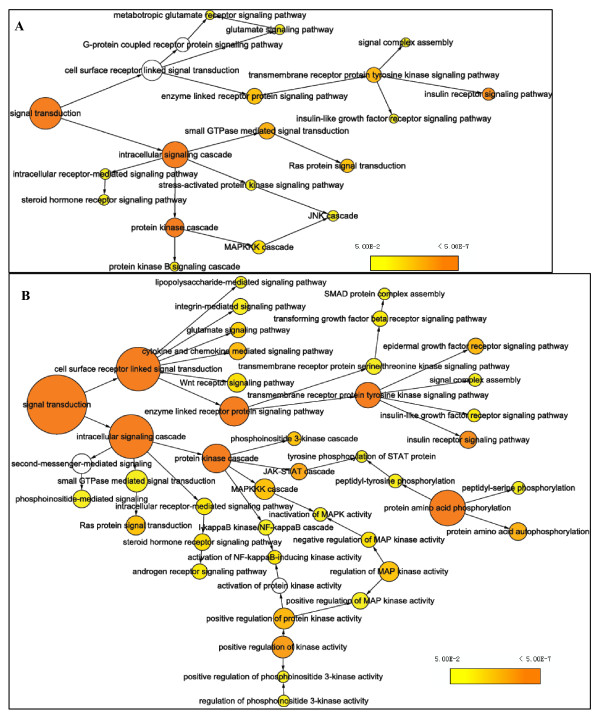
**Pathway cluster analysis for all node proteins from networks of Hep3B (A) and MHCC97H (B) cell lines**. Every ellipase node size represents the number of proteins assigned to each pathway; node color indicates correct p-value of each pathway from statistical test, orange denotes higher relative levels of overrepresented pathways with less p-value and yellow indicates lower levels of overrepresented pathways with bigger p-value, and white node denotes no change on a pathway.

On the other hand, pathway cluster analysis suggested that there was considerable overall alteration in signal transduction during HCC cell metastasis (*p *= 8.23E-10 to *p *= 4.20E-25). The maximum difference between the two cell lines was observed in the transmembrane receptor tyrosine kinase signaling pathway, cell surface receptor-linked signal transduction pathway, and protein kinase cascade (Figure 6). In particular, the difference was prominent in the epidermal growth factor receptor (EGFR) signaling pathway, cytokine- and chemokine-mediated signaling pathway, and JAK-STAT and phosphoinoside 3-kinase (PI3K) cascades. It should be noted that the JNK cascade has a negative relationship with HCC metastasis in signaling pathway clustering (overrepresented in the Hep3B cell line rather than in the MHCC97H cell line). Surprisingly, the regulatory pattern of protein amino acid phosphorylation in the two cell lines was completely reversed. In MHCC97H cells, autophosphorylation was upregulated, while in Hep3B cells, dephosphorylation was upregulated (details of pathway clustering were outlined in Table S5a, S5b in the additional file 3).

### Verification of the pTyr proteins

**Figure 7:**
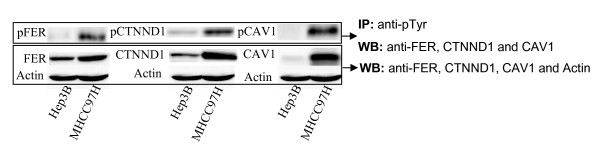
**FER, CTNND1, CAV1 and their phosphorylated version expression in two HCC cell lines**. First, equal protein lysates from two HCC cells lines were subjected to SDS-PAGE electrophoresis and subsequent Western blotting using anti-FER, CTNND1, CAV1 and actin antibodies (lower panel). Then in parallel experiment, equal protein lysates from two cell lines were immunoprecipitated using anti-pTyr 4G10 and pTyr100 antibodies, followed by reacting with FER, CTNND1 and CAV1 antibodies in Western blot assay (upper panel). This represents three independent experiments.

FER, CAV1, and CTNND1 were closely connected proteins in the MHCC97H network but were absent in the Hep3B network. These proteins were selected and tested for their expression in the two cell lines by using western blot assays. As shown in Figure 7, the total amount of these three proteins or the phosphorylated forms of these three proteins generally increased in the MHCC97H and Hep3B cell lines in response to their metastatic potential. This was consistent with the MS/MS results. These proteins were weakly expressed in Hep3B cells, and their amounts were below the detection limit of the MS assay.

**Figure 8:**
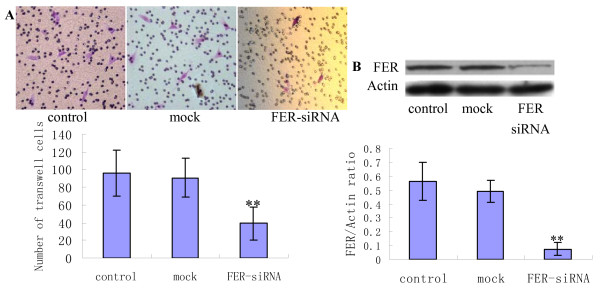
**Invasion activity reduction of MHCC97H cell by FER knockdown**. A: Matrigel invasion assay of MHCC97H cell. Image was viewed at ×200 and migrated cells were stained with purple, representing three independent experiments. B: Western blot assay of FER expression after siRNA in MHCC97H cell, representing three independent experiments. In comparison analysis, data was shown as Mean ± SD and statistics significance was calculated from the Student's test among every groups. Two asterisk denotes p < 0.01.

**Figure 9:**
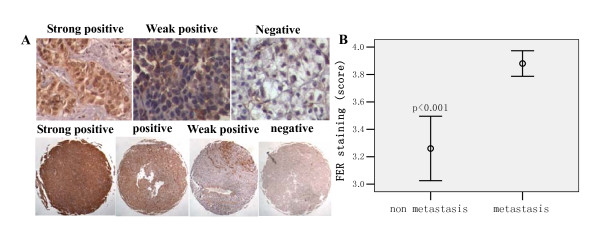
**Expression of FER in HCC tissue**. A: immunohistochemical staining of paraffin-embeded human HCC tissues showing different FER expression pattern in various samples (up panel is viewed at ×200 and low panel is viewed at ×50). B: comparison of FER staining in metastasis and non-metastatis HCC tissue (data was shown as the Mean ± SD).

The functions of FER were further verified by cell invasion experiments. FER expression in MHCC97H cells was knocked-down by treatment with a specific siRNA. The results showed that the invasive ability of treated MHCC97H cells was significantly weaker than that of the control cells, and only a few cells could pass though the matrigel-coated filter (Figure 8). This indicated that FER played a role in cell invasion in vitro. TMA analysis was performed to determine the expression level of FER in HCC tissues. The TMA was constructed from a total of 100 HCC cases, consisting of 50 liver cancer tissues each from patients with and without distant lung metastasis after liver cancer exairesis. TMA analysis showed that tumor tissue cells with distant pulmonary metastasis had higher FER expression levels than those without distant metastasis (*p *< 0.001, Figure 9). This was similar to the expression pattern observed in the cell lines.

## Discussion

Bioinformatics analysis based on computer calculations and the application of biological software is a very effective and essential supplement in proteomics research. It is a powerful tool that has been extensively used for MS/MS data analysis, such as PPI studies, functional clustering, pathway mapping, and prediction of protein function and structure [11,28-34]. In this study, we examined the expression profiles of pTyr proteins in two HCC cell lines (MHCC97H and Hep3B) that have different metastatic potentials. For this purpose, we used an LC-MS/MS-based proteomics technique. We also compared and analyzed the interactions and functions of these proteins by PPI and functional clustering analyses based on their GO attributes that were determined by biological process clustering and signaling pathway analysis. The results revealed that some cell functions related to cancer metastasis were significantly upregulated in HCC metastasis, especially those involved in cell motility, migration, localization, communication, antiapoptosis processes, and protein autophosphorylation. These alterations have been partly reported in previous studies on cancer metastasis [35-38]. High motility and migration are prerequisites for the successful metastasis of tumor cells and their transfer to the target site, penetration into the basement membrane and blood vessels, and movement from poorly oxygenated sites to rich oxygenated sites [3,39]. Antiapoptosis is a pivotal mechanism by which cancer cells are protected from elimination by means of various steps of a metastatic process [40]. At present, there are large amounts of data that highlight the importance of this process in cancer metastasis (reviewed in [40-43]). Protein amino acid autophosphorylation is a specific attribute of tyrosine kinase, and its upregulation in the highly metastatic MHCC97H cells confirmed that alteration of tyrosine phosphorylation was in fact involved in HCC metastasis. Western blot analysis of the pTyr protein profile also confirmed these results. More importantly, we found that alteration in cell communication was the most obvious cellular event in HCC metastasis. Since cell communication driven by signal transduction controls all cell behavior and activity, aberrations in signal transduction could be a crucial factor leading to HCC metastasis and could also trigger changes in cancer cell behavior. Therefore, it is very important to explore the events that occur during the activation and regulation of the signaling pathway in HCC metastasis. For this purpose, a pathway clustering analysis was performed for the node proteins of the PPI networks in Hep3B and MHCC97H cell lines. The results revealed that EGFR signal transduction, cytokine- and chemokine-mediated signaling, and the PI3K and JAK-STAT cascades were closely related with HCC metastasis since these processes were significantly upregulated. These analytical results were similar to those obtained for liver metastasis of breast cancer [11,29]. EGFR is an Erb-family transmembrane receptor tyrosine kinase. This receptor is shared by multiple ligands, including EGF, TGF, EGFF-like factor, and amphiregulin [44]. EGFR-mediated signaling has been implicated in a variety of human cancers and is a key regulator of the cell proliferation, migration, metastasis, angiogenesis, and antiapoptosis processes [45,46]. Recently, some studies have reported that EGFR inhibitors are effective first-line therapeutic agents in the treatment of some metastatic cancers [47,48]. EGFR is a potential therapeutic target in HCC treatment [49,50]. The JAK-STAT cascade, which is another example, is a critical signaling pathway that plays an important role in cell proliferation, differentiation, survival, motility, and apoptosis [51]. Recently, it was reported that sustained activation of the JAK-STAT cascade was involved in hepatocarcinogenesis and metastasis [52,53]. The data showed that increasing activity of the JAK-STAT cascade was associated with aberrant methylation silencing of the suppressor of cytokine signaling (SOCS), which is a negative regulator of the JAK-STAT pathway [54]. However, another study also suggested that constitutive activation of STAT3 was closely related to HCC metastasis and was the main factor leading to the upregulation of the JAK-STAT cascade [55]. Consistent with these results, our MS/MS results also indicated STAT3 activation. Unexpectedly, the JNK cascade was found to be negatively associated with HCC metastasis, thereby deviating from its canonical functions. This indicates that the role of this cascade in HCC metastasis should be re-evaluated. Negative regulation of the JNK cascade was recently reported in prostate and ovarian cancers [56,57]. Researchers showed that JNKK1, an upstream kinase of JNK, could persistently phosphorylate JNK and activate the stress-activated protein kinase signaling pathway (SAPK) to induce cell apoptosis and decrease tumor metastasis. Moreover, the SAPK pathway also showed a negative correlation with HCC metastasis in this study. Therefore, these two pathways might play synergetic roles in HCC metastasis. In other words, cluster analysis of signaling pathways may provide some important information for predicting the functions of the pTyr proteins involved in HCC metastasis. These results have been obtained by bioinformatics studies and need to be verified experimentally prior to practical application.

In our study, FER, a nonreceptor tyrosine kinase, was shown to be significantly involved in the HCC metastatic process. FER was initially discovered in 1988 during studies on the proto-oncogene protein Fes/Fps [58]. Since then, its involvement has been reported in growth factors/cytokine-mediated signaling as well as in the integrin/E-cadherin-mediated signaling pathways. It also plays a critical role in cytoskeletal regulation, cell adhesion, migration, and proliferation [59-61]. In this study, we examined the total and phosphorylated forms of FER; these were found to be overexpressed in the MHCC97H cell line but not in the Hep3B cell line. Similar expression patterns were observed for CTNND1 and CAV1, which are related to FER, and the results were also validated by western blotting and MS/MS analyses. FER and CTNND1 were shown to be involved in cadherin and integrin-mediated cell adhesion. Under normal circumstances, FER could indirectly sustain CTNNB1 dephosphorylation to ensure cadherin-mediated adhesion stability. When FER is overexpressed and phosphorylated, it can directly induce CTNNB1 and CTNND1 phosphorylation, resulting in the loss of cadherin-mediated adhesion [61,62]. This suggests that increasing metastasis may be associated with CTNNB1 in some cancers. Moreover, CTNND1 had been implicated in the metastasis and pathogenesis of several human cancers [63]. It was shown that overexpression and phosphorylation of CTNND1 in the cytoplasm could promote cadherin-deficient tumor metastasis by regulating the activity of the small GTPase [64,65]. CAV1 is an important marker protein of caveolae and regulates signal transduction as a scaffolding protein. Recently, CAV1 was found to be an independent predictor of decreased survival in breast and rectal cancers and was significantly associated with the presence of distant metastasis in colon cancer patients [66]. CAV1 could also sustain cadherin-mediated adhesion stability. It functioned by modulating the level and/or subcellular distribution of cadherin and CTNND1 by inhibiting Src kinase activity [67]. Nevertheless, FER was identified as a Src substrate and is involved in tumor transformation [59]. In this study, these three proteins displayed similar expression patterns in the two cell lines studied and also interacted directly in the MHHCC97H network. Therefore, we hypothesize that FER may be a key regulator in the adhesion event involving these three proteins and may participate in HCC metastasis. We also examined the influence of FER on cell invasion activity in vitro. The data illustrated that the knock-down of FER by RNAi significantly reduced MHCC97H cell invasion activity, suggesting that FER could positively contribute to HCC metastasis. To verify this, we further examined FER expression in HCC tumor tissues from patients with diverse distant metastasis outcomes after tumor excision. The results showed that FER overexpression frequently occurred in tumor cells with distant pulmonary metastasis, indicating that FER is strongly associated with HCC distant pulmonary metastasis.

Once it is confirmed that FER is a potential regulator in HCC metastasis, the next step is to understand the mechanism by which it regulates HCC cell metastasis. To date, there are few reports on the functional regulation of FER in cancer, and the main focus has been on cell adhesion. FER was shown to play a key role in the coordinate regulation of E-cadherin-mediated cell-cell adhesion and integrin-mediated focal adhesion. It could move between the two adhesion pathways to trigger adhesion transition [59,61]. In a study on FER function using the Trojan peptide, researchers showed that FER that had dissociated from the cadherin complex could translocate to the integrin complex where it reduced p130CAS phosphorylation and interrupted integrin-mediated focal adhesion by affecting the phosphorylation of p130CAS binding partners, including PTP-PEST, PTP1B, FAK, and Crk [62]. FER was also reported to be an upstream tyrosine kinase for CTTN (cortactin) as well as Src and Fyn [68,69]. Phosphorylation of CTTN by FER is very critical for its recruitment to the cadherin and integrin complex [70]. CTTN mainly participates in cytoskeletal regulation and plays a pivotal role in tumor metastasis [71,72]. Recently, CTTN was shown to be an essential regulator of matrix metalloproteinase secretion and extracellular matrix degradation [73]. In another study, it was shown that phosphorylation of tyrosine residues in CTTN was strongly associated with the potential to induce metastasis in nude mice [74]. Whether CTTN phosphorylation by FER also has the same effects in HCC metastasis needs to be confirmed in future studies.

The results of this study suggested that FER is involved in HCC distant metastasis, and we have discussed the main mechanism and signaling pathways associated with FER functional regulation. The results from bioinformatics analysis also imply that FER is altered in MHCC97H cells in comparison with Hep3B cells. As a new functional molecule, the effect of FER on HCC metastasis remains unknown. It is possible that FER can alter the metastatic potential of HCC cells by one or several of the mechanisms mentioned here. Further studies are required to explore the exact role of FER in HCC cell invasion and metastasis. These would be both exciting and challenging.

## Conclusion

The tyrosine phosphorylation data presented in this study is a useful resource for studying HCC metastasis and is an effective supplement for the PTyr database reported. Many of the identified pTyr proteins, including protein kinases and proteinases, could be useful for selecting intervention targets in future studies. The presence of multiple molecules and signaling pathways that are overrepresented in HCC cells with high metastatic potential suggests that HCC metastasis is a complex process and that the biological strategies for HCC metastasis treatment should be customized. In this study, FER was shown to be an important protein in HCC metastasis. We hope to obtain more evidence for the role of FER and its mechanism of action in HCC metastasis. This molecule may be a new drug target in future HCC therapy.

## Competing interests

The authors declare that they have no competing interests.

## Authors' contributions

HYL, ZGR and YKL contributed to design, analysis, interpretation of data. HYL performed the experiment. XNK helped to analyze the MS/MS data. LZ gave a hand in Western blot assay. XFL participated in cell invasion assay. YFS involved in cell culture. YW and TCX constructed TMA. HYL drafted the manuscript, YKL and ZGR revised it critically and gave smart suggestion for important content. All authors read and approved the manuscript.

## Pre-publication history

The pre-publication history for this paper can be accessed here:

http://www.biomedcentral.com/1471-2407/9/366/prepub

## Supplementary Material

Additional file 1 **Supplemental Tables S1 -S3**. Table S1: Common tyrosine phosphorylated proteins and sites from MHCC97H and Hep3B cell lines. Legend: The data in this table show the detailed information of the common tyrosine phosphorylated proteins and sites identified by LC-MS/MS in MHCC97H and Hep3B cell lines. Table S2: Tyrosine phosphorylated proteins and sites from MHCC97H cell line. Legend: The data in this table show the detailed information of the differentially expressed tyrosine phosphorylated proteins and sites identified by LC-MS/MS in MHCC97H cell line. Table S3: Tyrosine phosphorylated proteins and sites from Hep3B cell line. Legend: The data in this table show the detailed information of the differentially expressed tyrosine phosphorylated proteins and sites identified by LC-MS/MS in Hep3B cell line. Click here for file

Additional file 2 **Supplemental Table S4**. Table S4: Functional classification and distribution of tyrosine phosphorylated proteins from Hep3B and MHCC97H cell. Legend: The data provided display the detailed information in response to function and distribution of tyrosine phosphorylated proteins, which were identified by LC-MS/MS in MHCC97H and Hep3B cell lines. Click here for file

Additional file 3 **Supplemental Table S5**. Table S5a: Pathway clustering for node proteins in PPI network of MHCC97H cell. Table S5b: Pathway clustering for node proteins in PPI network of Hep3B cell. Legend: The two tables summarize detailed information of pathway clustering results generated from BinGO program, which was run to explore the functional regulation of node proteins in PPI network of MHCC97H and Hep3B cell. Click here for file
