# Supplementary material for: Identification of tyrosine-phosphorylated proteins associated with metastasis and functional analysis of FER in human hepatocellular carcinoma cells
Source: BMC Cancer. 2009 Oct 16;9:366. doi: 10.1186/1471-2407-9-366 (PMC2770568; doi:10.1186/1471-2407-9-366)
Supplement: Additional file 1 — Supplemental Tables S1 -S3. Table S1: Common tyrosine phosphorylated proteins and sites from MHCC97H and Hep3B cell lines. Legend: The data in this table show the detailed information of the common tyrosine phosphorylated proteins and sites identified by LC-MS/MS in MHCC97H and Hep3B cell lines. Table S2: Tyrosine phosphorylated proteins and sites from MHCC97H cell line. Legend: The data in this table show the detailed information of the differentially expressed tyrosine phosphorylated proteins and sites identified by LC-MS/MS in MHCC97H cell line. Table S3: Tyrosine phosphorylated proteins and sites from Hep3B cell line. Legend: The data in this table show the detailed information of the differentially expressed tyrosine phosphorylated proteins and sites identified by LC-MS/MS in Hep3B cell line. [file 1471-2407-9-366-S1.DOC]

**Table 1: Common tyrosine phosphorylated proteins and sites from MHCC97H and Hep3B cells.**

| Protein name | Accession NO. | Peptides and phosphorylated site | charge | Xcorr | DeltaCn |
| --- | --- | --- | --- | --- | --- |
| **RABL2A**  **SEPT2**  **TTN**  **WARS2**  **IRAK3**  **CCDC11**  **DPY19L2**  **KIAA1586**  **NEB**  **SESTD1**  **TIMELESS** | IPI00002644.1  IPI00014177.3  IPI00023283.3  IPI00025050.1  IPI00026984.1  IPI00065428.4  IPI00065441.5  IPI00259113.1  IPI00303335.1  IPI00329002.3  IPI00335541.4 | R.FLMDGFQPQQLpSTYALpTLp**Y**K.H  K.Ip**Y**HLPDAESDEDEDFKEQTR.L  K.AGREIp**Y**ESDKCSIR.S  R.MVWpSpTYSATVLTPGTTVTRLIEGNEYIFRVR.A  R.SSpTIKIIEFGQARQLK.P  R.pSSTIKIIEFGQARQLK.P  R.SpSTIKIIEFGQARQLK.P  K.Kp**Y**GEFFPVPEpSILTSM*KK.V  K.Kp**Y**GEFFPVPESILpTSM*KK.V  K.Kp**Y**GEFFPVPESILTpSM*KK.V  R.VEIQNLpTp**Y**AVKLFKQEK.K  K.LRELLALEENEp**Y**FTEMQLKK.E  K.VIFGILTVMSIQGp**Y**ANLR.N  K.IFIDKIYpSIYHQPNK.N  K.IFIDKIp**Y**SIYHQPNK.N  K.ALp**Y**pTLPRSVDDDPNpTAR.C  R.LTVPVVYPDGTEQYFGpSPpSDMApSTAENIRDRMK.L  R.LpTVPVVYPDGpTEQp**Y**FGSPSDMASTAENIRDRMK.L  R.FGGpSYIVQGLKSIGER.D  R.FGGSp**Y**IVQGLKSIGER.D | 3  3  2  3  2  2  2  2  2  2  3  3  2  2  2  2  3  4  2  2 | 2.78  2.95  2.04  2.66  2.28  2.21  2.21  2.94  2.65  2.43  2.56  2.81  2.35  2.13  2.08  2.01  3.62  2.86  2.79  2.76 | 0.13  0.56  0.59  0.71  0.58  0.58  0.68  0.68  0.63  0.73  0.21  0.62  0.61  0.63  0.65  0.15  0.18  0.60  0.32  0.51 |

**Table 2: Tyrosine phosphorylated proteins and sites** from MHCC97H cell.

| Protein name | Accession NO. | Peptides and phosphorylated site | charge | Xcorr | DeltaCn |
| --- | --- | --- | --- | --- | --- |
| **CAV2**  **ANXA2**  **PCBP2**  **CALM3**  **BCLAF1**  **SCRIB**  **ENSP00000369289**  **C14orf73**  **LRRC6**  **H-INV**  **MYLK2**  **KIAA1033**  **SERAC1**  **KIAA1383**  **SLC25A1**  **ENSP00000369289**  **FAM83A**  **FLJ34931**  **BRWD1**  **CTTN**  **USP14**  **FLJ90709**  **PCDHGB3**  **CAV1**  **PGRMC2**  **SF1**  **RASA2**  **CLCN6**  **SCGN**  **RB1**  **TFE3**  **C10orf71**  **DNASE1**  **CMPK**  **DNAH17**  **DAP3**  **DEGS2**  **RYR2**  **CRTAC1**  **SLC35F1**  **HOXD8**  **KCNB2**  **FRAP1**  **ZRANB3**  **ABLIM1**  **EPS8**  **PLEKHA5**  **MET**  **CTNND1**  **USP29**  **VCAM1**  **DST**  **GPR124**  **HIPK4**  **NT5DC1**  **ACOX1**  **SAMD14**  **ADPRH**  **SMG1**  **FAM13A1**  **NLRP11**  **BCOR**  **FLJ37396**  **GUCY2C**  **ULK2**  **APP**  **ANKRD11**  **CCDC112**  **LAMA3**  **GGPS1**  **FLJ41047**  **NXPH3**  **PADI1**  **SART3**  **DMXL1**  **SEPT9**  **LSP1**  **DICER1**  **MTMR8**  **MAGEB4**  **NBN**  **PIP5KL1**  **F2RL2**  **RP11-125A7.3**  **KIAA0256**  **HK2**  **GRIK3**  **MYH8**  **XPO1**  **ZDHHC23**  **RPS6KA1**  **WNT4**  **GSTK1**  **TXNRD1**  **RIF1**  **XIRP2**  **SERPINA10**  **ZNF746**  **MORF4L2**  **FER**  **STAT3**  **FEZ2**  **HNRNPU**  **PGRMC2**  **PRKCG**  **TRRAP**  **COL7A1**  **FMO3**  **KIAA1012**  **SNPH**  **C14orf28**  **ANXA1**  **DMRT2**  **CXXC5**  **EIF2C2**  **PER1**  **SLFN14**  **SLCO1B1**  **ANKRD31**  **SF1**  **FAT**  **ABCA2**  **TMEM2**  **ATIC**  **CYP8B1**  **BRUNOL5**  **C1RL**  **LAMA3**  **TNFSF10**  **ENO1**  **SLC10A3**  **PDK1**  **SORCS3**  **SNW1**  **OLFML1**  **RAD21**  **ATP11A**  **FAT3**  **FAM83B**  **FGD3**  **C6orf81**  **RASIP1**  **IFT140**  **RBL1**  **PTP4A2**  **7A5**  **ZFPM1**  **GPR116**  **SIGLEC8**  **COPB1**  **MLF2**  **DOCK3**  **TRIM26** | IPI00019870.1  IPI00418169.3  IPI00012066.2  IPI00075248.1  IPI00006079.1  IPI00410666.1  IPI00747793.2  IPI00073442.7  IPI00180190.3  IPI00455023.5  IPI00221127.3  IPI00164930.5  IPI00014444.6  IPI00847373.1  IPI00294159.3  IPI00747793.2  IPI00063301.2  IPI00397879.2  IPI00250716.1  IPI00029601.4  IPI00219913.10  IPI00168340.3  IPI00003893.1  IPI00009236.5  IPI00005202.2  IPI00294627.3  IPI00015811.1  IPI00639914.1  IPI00008730.2  IPI00302829.5  IPI00019490.1  IPI00427808.5  IPI00031065.1  IPI00219953.5  IPI00445211.1  IPI00018120.1  IPI00410145.2  IPI00023217.1  IPI00451624.1  IPI00299619.8  IPI00008481.1  IPI00024330.2  IPI00031410.1  IPI00030279.5  IPI00747332.3  IPI00290337.8  IPI00029515.3  IPI00884102.1  IPI00219872.1  IPI00011245.1  IPI00018136.1  IPI00074148.4  IPI00292834.5  IPI00302130.2  IPI00177965.5  IPI00296907.4  IPI00217977.2  IPI00009258.1  IPI00556369.3  IPI00006680.2  IPI00168280.1  IPI00100291.1  IPI00166296.6  IPI00012491.1  IPI00479399.1  IPI00412568.3  IPI00002286.5  IPI00746232.2  IPI00377045.3  IPI00032892.1  IPI00787788.1  IPI00028864.7  IPI00008040.4  IPI00006025.1  IPI00294728.1  IPI00455033.5  IPI00013260.3  IPI00219036.5  IPI00153020.1  IPI00006737.1  IPI00299463.1  IPI00161706.7  IPI00010870.4  IPI00158296.3  IPI00004067.2  IPI00102864.3  IPI00011397.1  IPI00302329.1  IPI00298961.3  IPI00217823.4  IPI00017305.2  IPI00011028.2  IPI00219673.6  IPI00554786.4  IPI00293845.4  IPI00550232.2  IPI00007199.4  IPI00071929.4  IPI00014174.1  IPI00029263.2  IPI00306436.1  IPI00006098.2  IPI00479217.1  IPI00005202.2  IPI00007128.1  IPI00069084.2  IPI00025418.2  IPI00329033.7  IPI00007253.3  IPI00006054.1  IPI00043479.4  IPI00218918.5  IPI00334605.2  IPI00152156.6  IPI00220349.5  IPI00440484.2  IPI00455330.3  IPI00295376.3  IPI00740057.2  IPI00386117.3  IPI00031411.3  IPI00307592.4  IPI00170706.2  IPI00289499.3  IPI00009440.1  IPI00171157.4  IPI00009793.3  IPI00790908.1  IPI00000049.3  IPI00465248.5  IPI00012852.3  IPI00014831.1  IPI00010381.2  IPI00013830.1  IPI00394820.3  IPI00006715.3  IPI00255653.4  IPI00455386.6  IPI00304527.4  IPI00384357.2  IPI00302301.2  IPI00015805.2  IPI00642186.1  IPI00005139.2  IPI00020191.1  IPI00376087.3  IPI00217346.2  IPI00437186.1  IPI00171647.1  IPI00295851.4  IPI00023095.1  IPI00217985.1  IPI00010948.2 | K.ADVQLFM*DDDSYSHHpSGLEYADPEK.F  K.ADVQLFM*DDDSYSHHSGLEp**Y**ADPEK.F  K.ADVQLFM*DDDSYpSHHSGLEYADPEK.F  K.ADVQLFM*DDDSp**Y**SHHSGLEYADPEK.F  K.ADVQLFM*DDDpSYpSHHSGLEYADPEK.F  R.RAEDGpSVIDYELIDQDAR.D  R.RAEDGSVIDp**Y**ELIDQDAR.D  K.GVTIPp**Y**RPKPSSSPVIFAGGQDR.Y  K.GVpTIPYRPKPSSSPVIFAGGQDR.Y  R.VFDKDGNGp**Y**ISAAELR.H  R.VFDKDGNGYIpSAAELR.H  K.LKDLFDp**Y**SPPLHK.N  R.p**Y**SRSLEELLLDANQLR.E  R.YpSRSLEELLLDANQLR.E  R.YSRpSLEELLLDANQLR.E  K.LTYTPIFNEp**Y**LSLVEK.Y  K.LpTYTPIFNEYLSLVEK.Y  K.LTp**Y**TPIFNEYLSLVEK.Y  R.HLETLIRpSYPDIRR.D  R.HLETLIRSp**Y**PDIRR.D  K.LKKLEp**Y**LNLALNNIEK.I  K.LFILp**Y**LQpTGDDFFPK.D  K.SQILLKKp**Y**LM*K.R  K.TTMNLp**Y**MSMQKPM*TK.T  R.p**Y**LLFPSLEVK.E  K.KNQIENp**Y**KEDK.Y  R.VCLDVAIVFVIp**Y**DEVVKLLNK.V  K.LTYTPIFNEp**Y**LSLVEK.Y  K.LTp**Y**TPIFNEYLSLVEK.Y  K.LpTYTPIFNEYLSLVEK.Y  K.LTYpTPIFNEYLSLVEK.Y  K.SSATVp**Y**FQpTVKHNNIRDLVR.R  K.SSApTVp**Y**FQTVKHNNIRDLVR.R  K.pSpSATVYFQTVKHNNIRDLVR.R  K.REPQEQPNLLQQLLQYTVpSK.L  K.REPQEQPNLLQQLLQp**Y**TVSK.L  K.Ip**Y**pSMTLRLSALFEEK.M  R.LPpSSPVYEDAASFK.A  R.LPSSPVp**Y**EDAASFK.A  R.ApSGEMASAQYITAALR.D  R.ASGEMApSAQYITAALR.D  R.ASGEMASAQp**Y**ITAALR.D  K.pSPSFFpSKFNILGTVSVLp**Y**LIFLVTFK.A  K.pSPpSFFSKFNILGTVSVLp**Y**LIFLVTFK.A  K.SPpSFFpSKFNILGTVSVLp**Y**LIFLVTFK.A  K.pSPpSFFSKFNILGTVpSVLYLIFLVTFK.A  R.ELSSp**Y**VSVSARpSGVVFAQRAFDHEQLR.A  R.ELpSSYVSVSARpSGVVFAQRAFDHEQLR.A  K.p**Y**VDSEGHLYTVPIR.E  K.YVDpSEGHLYTVPIR.E  K.YVDSEGHLp**Y**TVPIR.E  K.YVDSEGHLYpTVPIR.E  R.LLKPGEEPSEp**Y**TDEEDTKDHNKQD.-  R.LLKPGEEPpSEYTDEEDTKDHNKQD.-  R.LLKPGEEPSEYTDEEDpTKDHNKQD.-  R.TGDLGIPPNPEDRpSPpSPEPIYNSEGK.R  R.TGDLGIPPNPEDRpSPSPEPIp**Y**NSEGK.R  R.TGDLGIPPNPEDRpSPSPEPIYNpSEGK.R  K.M*EEACGpTIAVp**Y**QGPQKEPDDp**Y**SNFVIEDSVTTFK.T  R.LFTQLKFGVVQpTYK.F  R.LFTQLKFGVVQTp**Y**K.F  K.RDFEKIFAp**Y**p**Y**DVSK.T  R.p**Y**EEIp**Y**LKNKDLDAR.L  R.LFPHPPM*RLPRp**Y**LGR.G  K.RVKpSpTYSpSSPLLK.V  K.RVKSpTp**Y**SpSSPLLK.V  K.IAAFNIQTFGETKM*pSNATLVSp**Y**IVQILpSR.Y  K.IAAFNIQTFGETKM*pSNApTLVSYIVQILpSR.Y  K.IAAFNIQTFGETKM*SNApTLVSp**Y**IVQILpSR.Y  K.p**Y**IKEGKIVPVEIpTIpSLLK.R  R.LCRTYLAEp**Y**IR.T  R.p**Y**LLYGEKGTGK.T  K.EILAKp**Y**PAIK.A  R.LLpSLVEKVTp**Y**LK.K  R.FGAFARGAKVVLp**Y**pTK.K  K.YMILGLIDLEANp**Y**LVVK.A  -.M*SSYFVNPLp**Y**pSKp**Y**K.A  R.HPGAFpTSILNFp**Y**R.T  K.VTEDLpSKTLLMYTVPAVQGFFR.S  K.VTEDLSKpTLLMYTVPAVQGFFR.S  K.VpTEDLSKTLLMYTVPAVQGFFR.S  K.VTEDLSKTLLMp**Y**TVPAVQGFFR.S  K.LLEDGACVPFLNPp**Y**pTVQADLTVK.P  R.TSSESIp**Y**SRPGSSIPGSPGHTIp**Y**AK.V  R.LSTEHSSVSEp**Y**HPADGp**Y**AFSSNIYTR.G  R.AKpSPTPESSTIASp**Y**VTLR.K  R.SVSPTTEMVSNESVDp**Y**R.A  R.SMGp**Y**DDLDYGMMSDYGTAR.R  R.HYEDGYPGGSDNYGSLpSR.V  R.LHpSGp**Y**IFFp**Y**MHNGIFEELLR.K  K.DYFSPELLVLp**Y**FASpSLIIPAIGMIIYFARKANM*K.G  K.DYFSPELLVLYFApSpSLIIPAIGMIIYFARKANM*K.G  K.PQp**Y**EQLTAAGQGILSRPGEDPpSLRGIVK.E  K.PQYEQLpTAAGQGILpSRPGEDPSLRGIVK.E  R.pTLAGITAp**Y**QSCLQYPFTSVPLGGGAPGTRASR.R  R.TLAGITAp**Y**QpSCLQYPFTSVPLGGGAPGTRASR.R  K.PPpTPVVAAEDGpTPp**Y**YCLAEEK.E  K.PPpTPVVAAEDGTPp**Y**p**Y**CLAEEK.E  R.SGKYYFYDNYFDLPGALLCARVVDp**Y**LTK.L  R.IQPQQVAVWPTM*VDINSPESLTEAp**Y**KLR.A  R.p**Y**RPLHNAASHEGLAAApSCpSPPR.S  -.MEKpYVAAM*VLSAAGDALGp**Y**YNGK.W  K.LLYLCATp**Y**KALEpTVGEK.K  K.LLYLCApTYKALEpTVGEK.K  R.Fp**Y**AYGQpSRQYLDDTEVPPpSPPNSHSFMR.R  R.FYAp**Y**GQpSRQYLDDTEVPPSPPNSHpSFMR.R  R.CVGFTEADVSVLQAANILLPpSNTHKDRp**Y**K.F  K.YSLNMYKALLPQQSp**Y**pSLAQPLYpSPVCTNGER.F  K.QLIpSINNSCPApSKNVp**Y**LLEFHPSLSLAALIPK.A  K.EFQDILM*DHNRKSNVIIM*CGGPEFLp**Y**K.L  R.FASPPSLPDMQHIQEENLSSPPLGPPNp**Y**LQVSKDSASTSSK.N  R.FASPPSLPDMQHIQEENLSpSPPLGPPNYLQVSKDSASTSSK.N  R.VPTTAApSTPDAVDKp**Y**LETPGDENEHAHFQKAK.E  R.VPTpTAASTPDAVDKp**Y**LETPGDENEHAHFQKAK.E  K.M*EVKSYpTKNNTIAPK.K  K.M*EVKSYTKNNpTIAPK.K  K.M*EVKSp**Y**TKNNTIAPK.K  K.M*EVKpSYTKNNTIAPK.K  R.LIYEELIKEEKTpTNNELSAIpSR.K  R.LIp**Y**EELIKEEKTTNNELSAIpSR.K  K.YLTTADSpSLLQTNIALQLMEKSQKEYEK.L  K.YLTTADpSSLLQTNIALQLMEKSQKEYEK.L  K.p**Y**LTTADSSLLQTNIALQLMEKSQKEYEK.L  K.LRRGFPVAHSIYGIPpSVINpSANp**Y**VYFLGLEK.V  -.M*RKM#NTLLLVSLSFLp**Y**LK.E  -.M*RKM#NTLLLVSLpSFLYLK.E  K.VKKIFGWGDFYpSNIK.T  K.VKKIFGWGDFp**Y**SNIK.T  K.FLEDMpSYLTLKANCK.L  K.FLEDMSp**Y**LTLKANCK.L  R.YpSQp**Y**LDRQLKVK.D  R.p**Y**SQp**Y**LDRQLKVK.D  K.STVDVAFQDPSAVp**Y**SELILWRVDPVGPLpSFpSGGVSELAR.I  R.EMIPFAVVGpSDHEp**Y**QVNGK.R  K.IDQWLEQp**Y**pTQAIETAGRTPKLAR.Q  K.PIPQIPHFPVp**Y**TRSGEVTIpSIELK.K  K.PIPQIPHFPVYpTRSGEVTIpSIELK.K  K.LNAMANRAAGKGp**Y**ENEDNYANIR.F  K.M#LVQFLLp**Y**KYKM#K.E  K.AVESKKQPPQIEpSFp**Y**PPLDEPSIGSK.N  R.LLPDAPNALHILDGPEQRYFLGVVDLApTVp**Y**GLR.K  K.HTYALVTCGLVWATVFLp**Y**MLPFFILK.Q  R.SGM*QKLLCPVETHHIDIKGPALINIQEp**Y**PIER.H  R.KAp**Y**KDMVAAMEQEQAEEALK.N  R.FLRSEDGSGKGAAMVTAVAp**Y**R.L  K.ELpSNILGFIp**Y**DVKLVPDGKYGAQNDK.G  K.GKAEAHFSLIHYAGTVDp**Y**NITGWLDKNK.D  K.LISTLIp**Y**KFLNVPMFRNVpSLK.C  K.EQTLFFLSLGLFSLGYM*p**Y**p**Y**VFLQEVVPKGR.V  K.CNAVLKWp**Y**QKK.T  R.EAAFVp**Y**AISSAGVAFAVTR.A  K.PPGLLPRKGLp**Y**M*ANDLK.L  K.Ip**Y**SAERFLIATGERPR.Y  R.IIYIITVMVDCIDFpSPYNIKYQPKVK.S  R.IIYIITVMVDCIDFSPp**Y**NIKYQPKVK.S  K.REp**Y**AVHIAM*ENNLEK.V  K.LPp**Y**QGNApTM#LVVLMEK.M  K.HVM*RGNYETLVSLDp**Y**AISKPEVLSQIEQGK.E  R.IGAMLAp**Y**pTPLDEKSLALLLGYLHDFLKp**Y**LAK.N  K.KpSp**Y**IGVHQQIEAEMIK.V  R.CGNGGRANCDApSLIVTEELHLIpTFETEVp**Y**HQGLK.I  K.NERSHMPGpTYLpTTVIPp**Y**EKK.N  R.AKpSPQPPVEEEDEHFDDTVVCLDTYNCDLHFK.I  R.AKSPQPPVEEEDEHFDDpTVVCLDTYNCDLHFK.I  R.AKSPQPPVEEEDEHFDDTVVCLDpTYNCDLHFK.I  R.AKSPQPPVEEEDEHFDDTVVCLDTp**Y**NCDLHFK.I  R.LLKPGEEPSEp**Y**TDEEDTKDHNKQD.-  R.LLKPGEEPSEYTDEEDpTKDHNKQD.-  R.LLKPGEEPpSEYTDEEDTKDHNKQD.-  R.TFCGpTPDYIAPEIIAYQPYGK.S  R.TFCGTPDp**Y**IAPEIIAYQPYGK.S  R.FRpTATGAIpSAVFGRSQSLPGADSLLAK.P  K.YFTKEM*TAEFp**Y**ALKGM*FLAQINK.S  R.ERVTCTRLp**Y**AADIVFLLDGSS*SIGR.S  K.MLSFPLLLLAVTLpTFp**Y**.-  K.LFPHHIGELHILGVVp**Y**NLGTIQGSM*pTVDGIGALPGCHTGK.Y  R.RQGQPIp**Y**NIpSSLLR.G  K.YLLEGATLFNKEEHHp**Y**pSAAFQIGGHWMHp**Y**DGLR.N  K.AAp**Y**LQEpTGKPLDETLKK.A  K.CGPISDTLLp**Y**QQCLLNATTpSVQALKHGApSWDLK.G  K.QYAPVAYQNQVEp**Y**ENVARECRLGSK.E  K.SGNIPAGTTVDTKITHPTEFDFp**Y**LCpSHAGIQGTpSR.P  K.SGNIPAGTTVDTKITHPTEFDFYLCpSHAGIQGpTpSR.P  K.GRpSGTLApTLQp**Y**ALACVK.Q  K.EMLPHYVS*AFANpTQGGp**Y**VLIGVDDKSKEVVGCK.W  K.DQTANLTNQGKNITKNVTGFFQpSFK.S  K.DQTANLTNQGKNITKNVpTGFFQSFK.S  K.DQTANLTNQGKNIpTKNVTGFFQSFK.S  R.Vp**Y**LGLSSM#LRVSSLVLYIILIp**Y**AMKK.K  K.ASLFINKEDVp**Y**EYp**Y**QKDPK.N  K.ASLFINKEDVp**Y**Ep**Y**YQKDPK.N  R.TGDLGIPPNPEDRpSPpSPEPIYNSEGK.R  R.TGDLGIPPNPEDRpSPSPEPIp**Y**NSEGK.R  R.TGDLGIPPNPEDRpSPSPEPIYNpSEGK.R  K.LLDRETIpSGp**Y**TLTVQASDNGpSPPR.V  K.LLDREpTISGp**Y**TLTVQASDNGpSPPR.V  K.LLDRETIpSGYpTLTVQASDNGpSPPR.V  K.TTTMSILTGLFPPTpSGSApTIYGHDIRpTEMDEIR.K  K.TTTMSILTGLFPPTpSGSATIp**Y**GHDIRpTEMDEIR.K  K.TTTMSILTGLFPPTpSGpSATIYGHDIRpTEMDEIR.K  K.FYFDSSTGLLFLp**Y**LKAK.S  K.FYFDSSpTGLLFLYLKAK.S  K.FYFDpSSTGLLFLYLKAK.S  K.VCMVYDLp**Y**KpTLTPISAAp**Y**AR.A  K.VCMVYDLp**Y**KpTLTPIpSAAYAR.A  R.MRpTKHGDVFpTVQLGGQYFpTFVMDPLSFGPILK.D  R.MRpTKHGDVFpTVQLGGQp**Y**FTFVMDPLSFGPILK.D  K.GCAFLpTp**Y**CARDSAIKAQpTALHEQK.T  K.VLpSYVDWIK.G  K.VLSp**Y**VDWIK.G  R.IYQFARLNp**Y**TKGATSpSK.P  K.p**Y**pTSYPDPILLM*KSAR.N  K.IHAREIFDpSRGNPTVEVDLFpTSK.G  K.EGLELLKTAIGKAGp**Y**pTDK.V  R.RLQADp**Y**ASQAPFIVALSGTSEM*LALVIGHFIYpSSLFPVP.-  R.VEpTpSRAVPLAGFGp**Y**GLPISR.L  K.QFLDFGpSVNACEKTSFM*FLRQELPVR.L  K.VGLKpTVLSp**Y**LYVNPTNKR.K  K.p**Y**DAIARQGQSKDK.V  K.QILTLSWQGTGQVIp**Y**K.G  R.pTpSGHLLLGVVRIp**Y**HR.K  K.RpSAVEKpSMNAFLIVp**Y**LCILISK.A  R.FEKEVp**Y**DVSISEFpSPPGVVVAIVK.L  K.RHpSYAGEQPETVPp**Y**LLLNR.A  K.NGTPQDRHLFLFNpSM*ILpYCVPK.L  R.KSVVpSLATGAGAIp**Y**LLp**Y**KAIK.A  R.PYFLLLQGp**Y**QDAQDFVVYVMpTR.E  K.MGSHEGLLFFVpSLMDGpTVHp**Y**VDEKGK.T  R.pSALLp**Y**KFNGSPpSKSLK.D  R.GAFNpSKQLLp**Y**LEKYR.P  K.LTYIp**Y**SVVLTLVSEK.V  R.FSRHETp**Y**pTVHKR.Y  K.TDLETAFRKGp**Y**GILPGFK.G  K.WpSp**Y**KSQLNYKTK.Q  K.pSVKIp**Y**RGALWILGEYCpSpTK.E  R.QHMSRMLSGGFGp**Y**pSPFLSIpTDGNMPGTRPASR.R  R.p**Y**QEAFFDKDp**Y**INKHPGDAEK.I  K.GFTWGKVp**Y**WEVEVER.E | 3  3  3  3  3  2  2  3  3  2  2  2  2  2  2  2  2  2  2  2  2  2  2  2  2  2  3  2  2  2  2  3  3  3  2  2  2  2  2  2  2  2  4  4  4  4  3  3  2  2  2  2  3  3  3  3  3  3  4  3  3  2  2  2  3  3  3  3  3  3  2  2  2  2  2  3  2  2  3  3  3  3  3  3  3  2  2  2  2  3  5  5  4  4  3  3  3  3  5  4  3  4  3  3  3  3  4  4  5  4  5  5  4  4  2  2  2  2  4  4  4  4  4  4  3  3  2  2  2  2  2  2  4  3  4  4  4  3  2  4  4  3  4  3  3  3  5  3  4  2  2  2  2  3  3  2  2  5  3  2  4  4  4  4  3  3  4  4  3  2  2  4  4  5  3  5  4  2  5  3  6  6  2  5  3  3  3  4  2  2  3  3  3  3  3  3  5  5  5  2  2  3  3  3  3  3  3  2  2  2  2  3  2  5  2  2  2  2  2  3  4  3  5  3  4  3  4  2  2  2  4  3  3  2  3  3  2 | 5.66  4.71  4.65  4.26  5.69  6.39  4.39  3.38  2.93  5.08  4.29  3.04  2.85  2.94  2.94  3.00  2.96  2.82  2.17  2.15  2.10  2.59  2.15  2.01  2.03  2.04  3.69  3.07  2.13  3.00  2.09  3.04  2.54  2.51  2.47  2.28  2.20  3.90  3.18  4.45  3.33  2.61  2.88  2.88  2.81  2.76  2.62  2.58  2.23  2.14  2.77  2.29  5.00  4.81  3.87  3.23  2.83  2.68  2.68  2.62  2.60  2.11  2.10  2.07  2.59  2.55  2.57  3.10  2.61  2.77  2.24  2.22  2.08  2.01  2.00  2.74  2.12  2.08  2.86  2.82  2.74  2.67  3.76  4.15  5.09  4.44  2.86  2.88  3.77  2.63  2.63  2.54  2.66  2.82  2.89  2.59  2.75  2.72  3.01  2.84  2.53  2.60  2.92  2.90  3.13  2.81  2.73  2.99  2.84  2.63  2.91  2.55  2.64  2.57  2.07  2.05  2.03  2.03  2.50  2.68  3.35  3.14  3.03  3.05  2.69  2.58  2.14  2.01  2.11  2.10  2.08  2.01  2.75  2.71  2.68  2.65  2.62  2.52  2.39  3.02  2.81  2.70  2.67  2.64  2.63  2.60  2.53  2.52  2.52  2.23  2.08  2.12  2.11  2.59  2.50  2.11  2.01  2.52  2.51  2.04  2.81  2.75  6.78  4.82  4.04  3.01  4.38  3.69  4.81  4.95  3.86  2.64  2.79  2.64  2.57  2.69  2.18  2.73  2.40  2.76  2.78  2.80  2.64  2.01  2.59  2.85  2.73  2.62  2.72  2.17  2.15  3.23  3.10  2.68  2.98  2.90  2.87  2.65  2.54  2.70  2.14  2.89  2.56  3.37  2.88  2.68  2.56  2.67  2.09  2.03  2.03  2.07  2.57  2.10  2.70  2.13  2.60  2.19  2.20  2.02  3.12  2.85  2.75  2.56  2.65  2.61  2.59  2.55  2.15  2.10  2.09  2.80  2.58  2.53  2.06  2.50  2.54  2.33 | 0.17  0.18  0.25  0.25  0.13  0.34  0.64  0.21  0.52  0.16  0.62  0.54  0.63  0.28  0.46  0.42  0.45  0.63  0.41  0.64  0.13  0.44  0.53  0.66  0.44  0.22  0.22  0.43  0.53  0.44  0.54  0.48  0.49  0.53  0.11  0.30  0.52  0.26  0.58  0.25  0.41  0.60  0.39  0.41  0.42  0.43  0.43  0.45  0.19  0.12  0.17  0.40  0.10  0.23  0.48  0.13  0.17  0.35  0.11  0.40  0.46  0.15  0.15  0.10  0.29  0.38  0.18  0.16  0.17  0.36  0.17  0.20  0.31  0.14  0.29  0.59  0.12  0.19  0.58  0.59  0.60  0.71  0.21  0.12  0.11  0.10  0.23  0.11  0.10  0.23  0.14  0.28  0.30  0.14  0.17  0.18  0.24  0.26  0.25  0.27  0.20  0.24  0.40  0.45  0.16  0.18  0.17  0.13  0.15  0.52  0.12  0.13  0.49  0.50  0.57  0.58  0.58  0.60  0.45  0.49  0.57  0.59  0.62  0.48  0.51  0.53  0.55  0.55  0.50  0.51  0.42  0.44  0.10  0.24  0.13  0.55  0.57  0.15  0.14  0.34  0.62  0.62  0.68  0.64  0.36  0.57  0.24  0.63  0.69  0.51  0.62  0.66  0.64  0.11  0.14  0.54  0.11  0.18  0.71  0.67  0.11  0.18  0.29  0.58  0.55  0.64  0.17  0.19  0.23  0.26  0.60  0.56  0.40  0.18  0.21  0.53  0.12  0.58  0.57  0.35  0.58  0.12  0.19  0.43  0.10  0.48  0.50  0.57  0.21  0.55  0.55  0.13  0.27  0.40  0.29  0.30  0.32  0.12  0.12  0.11  0.68  0.62  0.17  0.45  0.56  0.63  0.67  0.64  0.25  0.58  0.60  0.53  0.63  0.61  0.54  0.60  0.61  0.18  0.23  0.12  0.26  0.28  0.57  0.12  0.59  0.46  0.62  0.26  0.58  0.58  0.63  0.42  0.12  0.26  0.67  0.50  0.60  0.60 |

**Table 3: Tyrosine phosphorylated proteins and sites from Hep3B cell**

| Protein name | Accession NO. | Peptides and phosphorylated site | charge | Xcorr | DeltaCn |
| --- | --- | --- | --- | --- | --- |
| **MPHOSPH1**  **MRPS35**  **GOLGA4**  **GFM1**  **OR2I1P**  **AADAC**  **RAB31**  **PDPK1**  **LGI1**  **NSUN4**  **AP2M1**  **CNNM2**  **GIMAP2**  **ST3GAL5**  **UBE1L**  **MAGI3**  **TSPYL1**  **RPS6KA4**  **TRPC1**  **MAN2A2**  **PROX1**  **CLSTN3**  **SORL1**  **GPR128**  **APOB**  **EIF4E**  **CCDC138**  **HS6ST3**  **SESTD1**  **CLCA3**  **PES1**  **LRRC4C**  **MDN1**  **DIP2C**  **FOXA1**  **PGM1**  **PDE1C**  **MLH1**  **HTATSF1**  **TTLL4**  **MLL4**  **CXCR7**  **TAS2R8**  **RFT1**  **CHM**  **CECR1**  **RBED1**  **GBAS**  **PANK1**  **ZNF420**  **ZNF286A**  **CLK2**  **FAM12B**  **SFRS1**  **UROD**  **TRA2A**  **WARS2**  **TOP1MT**  **BRIP1**  **SACS**  **SOLU**  **MYO1F**  **CEBPB**  **XPNPEP2**  **ITPKB**  **PYHIN1**  **LCORL**  **HPSE2**  **HSF2BP**  **DUSP4**  **RBM15**  **FEM1B**  **FAM47B** | IPI00044751.5  IPI00073779.1  IPI00013272.1  IPI00154473.4  IPI00075660.3  IPI00383879.6  IPI00014376.5  IPI00002538.1  IPI00021091.1  IPI00303944.6  IPI00022256.3  IPI00006084.4  IPI00022139.4  IPI00761101.2  IPI00013183.7  IPI00170865.2  IPI00164215.4  IPI00022536.1  IPI00012081.1  IPI00027703.1  IPI00152167.3  IPI00396423.3  IPI00022608.1  IPI00064461.3  IPI00022229.1  IPI00027485.3  IPI00065415.2  IPI00218046.6  IPI00329002.3  IPI00783194.1  IPI00003768.1  IPI00014223.2  IPI00167941.1  IPI00032087.4  IPI00024387.1  IPI00844159.2  IPI00028928.1  IPI00029754.1  IPI00013788.1  IPI00005635.2  IPI00218823.3  IPI00012733.3  IPI00028325.1  IPI00059368.2  IPI00028099.2  IPI00252768.4  IPI00796589.2  IPI00016077.1  IPI00152578.2  IPI00154558.1  IPI00010163.1  IPI00028071.3  IPI00011596.1  IPI00215884.4  IPI00301489.3  IPI00013891.1  IPI00025050.1  IPI00465141.2  IPI00012500.1  IPI00646703.3  IPI00748177.1  IPI00218638.9  IPI00289773.3  IPI00439344.1  IPI00021449.3  IPI00103253.1  IPI00043716.4  IPI00029594.8  IPI00023897.1  IPI00011860.3  IPI00102752.2  IPI00033419.2  IPI00176156.5 | K.EVQQIQpSNp**Y**DIAIAELHVQK.S  K.M*LRLpSQDVKGp**Y**pSFIK.D  K.MAVDQDWPpSVYPVAAPFKPSAVPLPVRMGp**Y**PVK.K  K.MAVDQDWPpSVYPVAAPFKPpSAVPLPVRMGYPVK.K  K.MAVDQDWPSVp**Y**PVAAPFKPSAVPLPVRMGp**Y**PVK.K  R.IM*ELEDHIpTQKTIEIESLNEVLKNp**Y**NQQK.D  R.IM*ELEDHITQKTIEIEpSLNEVLKNp**Y**NQQK.D  R.IM*ELEDHITQKpTIEIESLNEVLKNp**Y**NQQK.D  K.TTLTERVLp**Y**YTGRIAK.M  K.TTLTERVLYp**Y**TGRIAK.M  K.FVpSLFYTVVTPALNPLIp**Y**pTLRNK.K  K.FVSLFYpTVVTPALNPLIp**Y**pTLRNK.K  K.FVSLFp**Y**TVVTPALNPLIp**Y**pTLRNK.K  R.GLPLTYVIpTCQYDLLRDDGLM*p**Y**VTRLR.N  R.GLPLTYVITCQp**Y**DLLRDDGLM*p**Y**VTRLR.N  K.Ep**Y**AESIGAIVVETSAKNAINIEELFQGISR.Q  K.EYAEpSIGAIVVETSAKNAINIEELFQGISR.Q  K.EYAESIGAIVVETSAKNAINIEELFQGISR.Q  K.LYFpTFQDDEKLYFGLpSYAK.N  K.LYFpTFQDDEKLYFGLSp**Y**AK.N  R.IAp**Y**FLCLLSALLLpTEGKK.P  R.IAYFLCLLpSALLLpTEGKK.P  R.LQKILHSp**Y**VPEEIR.D  R.LQKILHpSYVPEEIR.D  K.WVRYIGRSGIYEpTR.C  K.WVRYIGRSGIp**Y**ETR.C  K.FEApSAFSp**Y**YGVMALTASPVPLSLSRTFVVSR.T  K.FEApSAFpSYp**Y**GVMALTASPVPLSLSRTFVVSR.T  K.QSLIKp**Y**METQRSYTALAEANCLK.G  K.QSLIKYMEpTQRSYTALAEANCLK.G  R.AMPSEYpTYVKLR.S  R.AMPSEYTp**Y**VKLR.S  R.HSYLHLAENp**Y**LIRp**Y**M*PFAPAIQTFHHLK.W  R.Hp**Y**LpSLQFQKGSIDHKLQQVIR.D  R.Np**Y**IIQNIPGFWMTAFR.N  R.ITEANLTGHEEKVpSVENFELLKVLGpTGAp**Y**GK.V  K.PTCKKIMTVLTVGIFWPVLSLCp**Y**LIAPK.S  K.LPLQANFp**Y**PMPVMAYIQDAQK.R  K.LLKRANSp**Y**EDAM*MPFPGApTIIpSQLLK.N  R.AAVTEGKLp**Y**DRILR.V  R.NVVYGIFp**Y**ATSFLDLYR.N  R.LRVKMp**Y**NFLR.S  R.FLKNIILPVp**Y**DK.S  R.Vp**Y**KERLGLPPK.I  R.LDNLQRKp**Y**EFM*TIQR.L  R.QVRMLADLSLVGCp**Y**NLTFM*NESERNTILLQSAK.N  R.LpTVPVVp**Y**PDGTEQYFGSPSDMASpTAENIRDRMK.L  R.M*NQAAELp**Y**LIQIIEKGSLVGLVTFDpSFAKIQpSK.L  K.GSTAARTFp**Y**LIK.D  K.IIIGCFVAITLMAAVM*LVIFp**Y**K.M  R.ITPYDVQLGp**Y**pSVLSR.G  K.DVGLWHGILTpSVMNMMHVISIPp**Y**SLM*KVNPLSWIQK.V  K.RpSp**Y**PHAKPPYSYISLITMAIQR.A  K.FKPFTGILEIVDSVEAp**Y**ATMLR.S  K.FKPFTGILEIVDSVEAYApTMLR.S  K.FIFYELLpTRp**Y**DLISR.F  K.NpTHPFLp**Y**LpSLEISPQNVDVNVHPTK.H  R.EFDEDpSDEKEEEEDTYEK.V  K.VFDDEpSDEKEDEEYADEK.G  K.VFDDESDEKEDEEp**Y**ADEK.G  K.DLDEEGpSEKELHENVLDK.E  K.pTIISSEPp**Y**VTSLLKMYVR.R  K.pTIIpSSEPYVTSLLKMYVR.R  K.RNIDAGEM*VIEp**Y**pSGIVIR.S  K.p**Y**SAKTGLTKLIDASR.V  K.YpSAKTGLTKLIDASR.V  K.YSAKpTGLTKLIDASR.V  K.KKISTVDp**Y**ILpTNLVIAR.I  K.KKISpTVDYILpTNLVIAR.I  K.EIVGVVNVRLTLLYpSpTTLFLAREAFR.R  K.EIVGVVNVRLTLLp**Y**SpTTLFLAREAFR.R  R.FNIDLVSKLLp**Y**SR.G  R.FNIDLVSKLLYpSR.G  R.MAMGLRIKFPTVVAGFDLVGHEDpTGHSLHDp**Y**K.E  R.MAMGLRIKFPTVVAGFDLVGHEDpTGHpSLHDYK.E  R.GAGFLALLHLLYLVMDpSK.T  R.GAGFLALLHLLp**Y**LVMDSK.T  R.pSGPNIYELRSYQLR.P  R.SGPNIp**Y**ELRSYQLR.P  K.YLpTSNTAYGKTGIR.D  K.p**Y**LTSNTAYGKTGIR.D  R.DVM*LENYpSNLVSLDLPSR.C  R.DVM*LENp**Y**SNLVSLDLPSR.C  K.DVAM*DFpTPEEWGKLDPAQRDVM*LENp**Y**R.N  R.DRGDAYYDTDYRHpSYEYQR.E  R.DRGDAYYDTDYRHSYEp**Y**QR.E  K.QHp**Y**LpSPpSR.E  K.VDGPRpSPSp**Y**GRpSR.S  R.DPEVVASELGp**Y**VFQAIpTLpTRQR.L  R.RRS#PSPYYpSR.Y  R.RRSPpSPp**Y**YSR.Y  K.Kp**Y**GEFFPVPEpSILTSM*KK.V  K.p**Y**IMLNPCSKLK.G  K.TNFDELLQVp**Y**YDAIKYK.G  K.FLTTp**Y**HELIPSR.K  K.EQGYIVLTp**Y**LWDLK.I  K.CVp**Y**VIGREKMK.K  K.PAEp**Y**GYVSLGRLGAAK.G  R.DAVAVIRp**Y**LVWLEKNVPK.G  R.EFTKGNHNILIAp**Y**R.D  -.M*ANNp**Y**KKIVLLK.G  K.M*IRQFAIEp**Y**ISK.S  R.IYSRASLp**Y**GPNIGR.P  K.VLM*LMSLp**Y**NVSINLK.G  R.PFLAHSAGp**Y**ILGpSVNVR.C  R.pSRSPLDKDTp**Y**PPSASVVGASVGGHR.H  R.p**Y**LLGYVSQQGGQR.S  K.pTPIQRAVQVp**Y**K.Y | 2  2  4  4  4  4  4  4  3  3  2  2  2  3  3  4  4  4  3  3  4  4  2  2  2  2  3  3  3  3  2  2  4  3  2  3  4  3  3  2  2  2  2  2  2  4  4  4  3  2  2  6  3  3  3  2  3  2  2  2  2  3  3  2  2  2  2  3  3  3  3  2  2  4  4  2  2  2  2  2  2  2  2  3  4  4  2  2  3  2  2  3  2  2  2  2  2  2  2  2  2  2  2  2  2  3  2  2 | 2.99  2.46  2.89  2.83  3.25  3.05  3.38  3.54  3.20  2.61  2.23  2.16  2.13  2.81  2.50  3.96  3.47  3.30  3.48  3.42  2.59  2.72  2.43  2.41  2.32  2.19  2.64  2.51  2.95  2.85  2.05  2.03  3.33  2.82  2.38  2.79  3.09  2.57  2.56  2.01  2.31  2.17  2.12  2.06  2.02  2.57  3.07  2.64  2.70  2.17  2.02  2.62  2.76  2.64  2.62  2.09  2.60  3.50  6.19  3.22  4.36  3.11  3.05  2.59  2.14  2.23  2.00  3.13  2.59  2.81  2.79  2.78  2.73  2.75  2.59  2.44  2.22  2.35  2.13  2.20  2.12  2.12  2.01  3.28  3.16  2.83  2.27  2.45  3.25  2.39  2.07  2.83  2.77  2.09  2.21  2.41  2.34  2.29  2.29  2.26  2.06  2.06  2.00  2.05  2.08  3.22  2.07  2.04 | 0.49  0.49  0.35  0.42  0.30  0.36  0.41  0.46  0.27  0.42  0.43  0.44  0.49  0.14  0.20  0.33  0.32  0.24  0.44  0.53  0.32  0.17  0.53  0.70  0.35  0.48  0.62  0.66  0.59  0.61  0.11  0.14  0.57  0.20  0.22  0.52  0.46  0.61  0.56  0.13  0.52  0.63  0.52  0.46  0.21  0.18  0.57  0.13  0.18  0.59  0.61  0.13  0.51  0.62  0.65  0.69  0.62  0.47  0.48  0.50  0.49  0.46  0.49  0.68  0.57  0.54  0.58  0.28  0.32  0.59  0.63  0.41  0.48  0.46  0.47  0.52  0.64  0.45  0.56  0.50  0.59  0.64  0.69  0.15  0.14  0.21  0.38  0.29  0.57  0.12  0.30  0.54  0.34  0.59  0.39  0.21  0.42  0.64  0.64  0.68  0.61  0.62  0.18  0.61  0.44  0.21  0.59  0.49 |
