# Supplementary material for: Identification of tyrosine-phosphorylated proteins associated with metastasis and functional analysis of FER in human hepatocellular carcinoma cells
Source: BMC Cancer. 2009 Oct 16;9:366. doi: 10.1186/1471-2407-9-366 (PMC2770568; doi:10.1186/1471-2407-9-366)
Supplement: Additional file 2 — Supplemental Table S4. Table S4: Functional classification and distribution of tyrosine phosphorylated proteins from Hep3B and MHCC97H cell. Legend: The data provided display the detailed information in response to function and distribution of tyrosine phosphorylated proteins, which were identified by LC-MS/MS in MHCC97H and Hep3B cell lines. [file 1471-2407-9-366-S2.DOC]

**Table 4:** **Functional classification and distribution of tyrosine phosphorylated proteins from Hep3B and MHCC97H cell.**

| Protein name | Accession NO. | Molecule function | Biological process | Distribution | Liver or Liver cancer expression |
| --- | --- | --- | --- | --- | --- |
| **MHCC97H cell**  ***RABL2A***  ***SEPT2***  ***TTN***  ***WARS2***  ***IRAK3***  ***CCDC11***  ***DPY19L2***  ***KIAA1586***  ***NEB***  ***SESTD1***  ***TIMELESS***  *CAV2*  *ANXA2*  *PCBP2*  *CALM3*  *BCLAF1*  *SCRIB*  *ENSP00000369289*  *C14orf73*  *LRRC6*  *H-INV*  *MYLK2*  *KIAA1033*  *SERAC1*  *KIAA1383*  *SLC25A1*  *ENSP00000369289*  *FAM83A*  *FLJ34931*  *BRWD1*  *CTTN*  *USP14*  *FLJ90709*  *PCDHGB3*  *CAV1*  *PGRMC2*  *SF1*  *RASA2*  *CLCN6*  *SCGN*  *RB1*  *TFE3*  *C10orf71*  *DNASE1*  *CMPK*  *DNAH17*  *DAP3*  *DEGS2*  *RYR2*  *CRTAC1*  *SLC35F1*  *HOXD8*  *KCNB2*  *FRAP1*  *ZRANB3*  *ABLIM1*  *EPS8*  *PLEKHA5*  *MET*  *CTNND1*  *USP29*  *VCAM1*  *DST*  *GPR124*  *HIPK4*  *NT5DC1*  *ACOX1*  *SAMD14*  *ADPRH*  *SMG1*  *FAM13A1*  *NLRP11*  *BCOR*  *FLJ37396*  *GUCY2C*  *ULK2*  *APP*  *ANKRD11*  *CCDC112*  *LAMA3*  *GGPS1*  *FLJ41047*  *NXPH3*  *PADI1*  *SART3*  *DMXL1*  *SEPT9*  *LSP1*  *DICER1*  *MTMR8*  *MAGEB4*  *NBN*  *PIP5KL1*  *F2RL2*  *RP11-125A7.3*  *KIAA0256*  *HK2*  *GRIK3*  *MYH8*  *XPO1*  *ZDHHC23*  *RPS6KA1*  *WNT4*  *GSTK1*  *TXNRD1*  *RIF1*  *XIRP2*  *SERPINA10*  *ZNF746*  *MORF4L2*  *FER*  *STAT3*  *FEZ2*  *HNRNPU*  *PGRMC2*  *PRKCG*  *TRRAP*  *COL7A1*  *FMO3*  *KIAA1012*  *SNPH*  *C14orf28*  *ANXA1*  *DMRT2*  *CXXC5*  *EIF2C2*  *PER1*  *SLFN14*  *SLCO1B1*  *ANKRD31*  *SF1*  *FAT*  *ABCA2*  *TMEM2*  *ATIC*  *CYP8B1*  *BRUNOL5*  *C1RL*  *LAMA3*  *TNFSF10*  *ENO1*  *SLC10A3*  *PDK1*  *SORCS3*  *SNW1*  *OLFML1*  *RAD21*  *ATP11A*  *FAT3*  *FAM83B*  *FGD3*  *C6orf81*  *RASIP1*  *IFT140*  *RBL1*  *PTP4A2*  *7A5*  *ZFPM1*  *GPR116*  *SIGLEC8*  *COPB1*  *MLF2*  *DOCK3*  *TRIM26*  **Hep3B cell**  MPHOSPH1  MRPS35  GOLGA4  GFM1  OR2I1P  AADAC  RAB31  PDPK1  LGI1  NSUN4  AP2M1  CNNM2  GIMAP2  ST3GAL5  UBE1L  MAGI3  TSPYL1  RPS6KA4  TRPC1  MAN2A2  PROX1  CLSTN3  SORL1  GPR128  APOB  EIF4E  CCDC138  HS6ST3  SESTD1  CLCA3  PES1  LRRC4C  MDN1  DIP2C  FOXA1  PGM1  PDE1C  MLH1  HTATSF1  TTLL4  MLL4  CXCR7  TAS2R8  RFT1  CHM  CECR1  RBED1  GBAS  PANK1  ZNF420  ZNF286A  CLK2  FAM12B  SFRS1  UROD  TRA2A  WARS2  TOP1MT  BRIP1  SACS  SOLU  MYO1F  CEBPB  XPNPEP2  ITPKB  PYHIN1  LCORL  HPSE2  HSF2BP  DUSP4  RBM15  FEM1B  FAM47B | IPI00002644.1  IPI00014177.3  IPI00023283.3  IPI00025050.1  IPI00026984.1  IPI00065428.4  IPI00065441.5  IPI00259113.1  IPI00303335.1  IPI00329002.3  IPI00335541.4  IPI00019870.1  IPI00418169.3  IPI00012066.2  IPI00075248.1  IPI00006079.1  IPI00410666.1  IPI00747793.2  IPI00073442.7  IPI00180190.3  IPI00455023.5  IPI00221127.3  IPI00164930.5  IPI00014444.6  IPI00847373.1  IPI00294159.3  IPI00747793.2  IPI00063301.2  IPI00397879.2  IPI00250716.1  IPI00029601.4  IPI00219913.10  IPI00168340.3  IPI00003893.1  IPI00009236.5  IPI00005202.2  IPI00294627.3  IPI00015811.1  IPI00639914.1  IPI00008730.2  IPI00302829.5  IPI00019490.1  IPI00427808.5  IPI00031065.1  IPI00219953.5  IPI00445211.1  IPI00018120.1  IPI00410145.2  IPI00023217.1  IPI00451624.1  IPI00299619.8  IPI00008481.1  IPI00024330.2  IPI00031410.1  IPI00030279.5  IPI00747332.3  IPI00290337.8  IPI00029515.3  IPI00884102.1  IPI00219872.1  IPI00011245.1  IPI00018136.1  IPI00074148.4  IPI00292834.5  IPI00302130.2  IPI00177965.5  IPI00296907.4  IPI00217977.2  IPI00009258.1  IPI00556369.3  IPI00006680.2  IPI00168280.1  IPI00100291.1  IPI00166296.6  IPI00012491.1  IPI00479399.1  IPI00412568.3  IPI00002286.5  IPI00746232.2  IPI00377045.3  IPI00032892.1  IPI00787788.1  IPI00028864.7  IPI00008040.4  IPI00006025.1  IPI00294728.1  IPI00455033.5  IPI00013260.3  IPI00219036.5  IPI00153020.1  IPI00006737.1  IPI00299463.1  IPI00161706.7  IPI00010870.4  IPI00158296.3  IPI00004067.2  IPI00102864.3  IPI00011397.1  IPI00302329.1  IPI00298961.3  IPI00217823.4  IPI00017305.2  IPI00011028.2  IPI00219673.6  IPI00554786.4  IPI00293845.4  IPI00550232.2  IPI00007199.4  IPI00071929.4  IPI00014174.1  IPI00029263.2  IPI00306436.1  IPI00006098.2  IPI00479217.1  IPI00005202.2  IPI00007128.1  IPI00069084.2  IPI00025418.2  IPI00329033.7  IPI00007253.3  IPI00006054.1  IPI00043479.4  IPI00218918.5  IPI00334605.2  IPI00152156.6  IPI00220349.5  IPI00440484.2  IPI00455330.3  IPI00295376.3  IPI00740057.2  IPI00386117.3  IPI00031411.3  IPI00307592.4  IPI00170706.2  IPI00289499.3  IPI00009440.1  IPI00171157.4  IPI00009793.3  IPI00790908.1  IPI00000049.3  IPI00465248.5  IPI00012852.3  IPI00014831.1  IPI00010381.2  IPI00013830.1  IPI00394820.3  IPI00006715.3  IPI00255653.4  IPI00455386.6  IPI00304527.4  IPI00384357.2  IPI00302301.2  IPI00015805.2  IPI00642186.1  IPI00005139.2  IPI00020191.1  IPI00376087.3  IPI00217346.2  IPI00437186.1  IPI00171647.1  IPI00295851.4  IPI00023095.1  IPI00217985.1  IPI00010948.2  IPI00044751.5  IPI00073779.1  IPI00013272.1  IPI00154473.4  IPI00075660.3  IPI00383879.6  IPI00014376.5  IPI00002538.1  IPI00021091.1  IPI00303944.6  IPI00022256.3  IPI00006084.4  IPI00022139.4  IPI00761101.2  IPI00013183.7  IPI00170865.2  IPI00164215.4  IPI00022536.1  IPI00012081.1  IPI00027703.1  IPI00152167.3  IPI00396423.3  IPI00022608.1  IPI00064461.3  IPI00022229.1  IPI00027485.3  IPI00065415.2  IPI00218046.6  IPI00329002.3  IPI00783194.1  IPI00003768.1  IPI00014223.2  IPI00167941.1  IPI00032087.4  IPI00024387.1  IPI00844159.2  IPI00028928.1  IPI00029754.1  IPI00013788.1  IPI00005635.2  IPI00218823.3  IPI00012733.3  IPI00028325.1  IPI00059368.2  IPI00028099.2  IPI00252768.4  IPI00796589.2  IPI00016077.1  IPI00152578.2  IPI00154558.1  IPI00010163.1  IPI00028071.3  IPI00011596.1  IPI00215884.4  IPI00301489.3  IPI00013891.1  IPI00025050.1  IPI00465141.2  IPI00012500.1  IPI00646703.3  IPI00748177.1  IPI00218638.9  IPI00289773.3  IPI00439344.1  IPI00021449.3  IPI00103253.1  IPI00043716.4  IPI00029594.8  IPI00023897.1  IPI00011860.3  IPI00102752.2  IPI00033419.2  IPI00176156.5 | GTPase  GTPase  cytoskeletin  Translation regulatory protein  Serine and threonine kinase  unknown  unknown  unknown  Cytoskeletal protein  Cytoskeletal associated protein  Transcription regulatory protein  Integral membrane protein  Calcium binding protein  RNA binding protein  Calcium binding protein  Transcription factor  Ubiquitin proteasome system protein  unknown  unknown  unknown  unknown  Serine/threonine kinase  unknown  unknown  Unclassified  Transport /cargo protein  unknown  Unclassified  unknown  Unclassified  Cytoskeletal associated protein  Ubiquitin proteasome system protein  Unclassified  Adhesion molecule  Structural protein  Integral membrane protein  RNA binding protein  GTPase activating protein  Voltage gated channel  Calcium binding protein  Transcription regulatory protein  Transcription factor  Unclassified  Deoxyribonuclease  Enzyme: Phosphotransferase  Motor protein  Ribosomal subunit  Enzyme: Hydroxylase  Intracellular ligand gated channel  Calcium binding protein  Integral membrane protein  Transcription factor  Voltage gated channel  Cell cycle control protein  Unclassified  Cytoskeletal associated protein  Adapter molecule  Adapter molecule  Receptor tyrosine kinase  Adhesion molecule  Ubiquitin proteasome system protein  Adhesion molecule  Cytoskeletal associated protein  G protein coupled receptor  Serine/threonine kinase  Unclassified  Enzyme: Oxidase  Unclassified  Enzyme: Hydrolase  Unclassified  Unclassified  Unclassified  Transcription regulatory protein  unknown  Guanylate cyclase  Growth factor  Cell surface receptor  Transcription regulatory protein  Unclassified  Extracellular matrix protein  Enzyme: Prenyltransferase  unknown  Secreted polypeptide  Enzyme: Hydrolase  RNA binding protein  Unclassified  GTPase  Calcium binding protein  Ribonuclease  Lipid phosphatase  Unclassified  DNA repair protein  Adapter molecule  G protein coupled receptor  unknown  Unclassified  Enzyme: Phosphotransferase  Extracellular ligand gated channel  Structural protein  Transport/cargo protein  Unclassified  Serine/threonine kinase  Ligand  Enzyme: Glutathione transferase  Enzyme: Oxidoreductase  DNA binding protein  Unclassified  Protease inhibitor  Transcription regulatory protein  Transcription regulatory protein  Tyrosine kinase  Transcription factor  Unclassified  Ribonucleoprotein  Integral membrane protein  Serine/threonine kinase  Transcription regulatory protein  Extracellular matrix protein  Enzyme: Oxygenase  Transport/cargo protein  Unclassified  Unclassified  Calcium binding protein  Transcription regulatory protein  DNA binding protein  Translation regulatory protein  Transcription regulatory protein  unknown  Membrane transport protein  unknown  RNA binding protein  Adhesion molecule  Transport/cargo protein  Integral membrane protein  Enzyme: Hydrolase  Enzyme: Hydroxylase  RNA binding protein  Serine protease  Adhesion molecule  Ligand  Enzyme: Hydratase  Transport/cargo protein  Enzyme: Phosphotransferase  Cell surface receptor  Transcription regulatory protein  Unclassified  DNA binding protein  ATPase  Integral membrane protein  Unclassified  Guanine nucleotide exchange factor  Unclassified  Unclassified  Unclassified  Cell cycle control protein  Tyrosine phosphatase  Unclassified  Transcription regulatory protein  G protein coupled receptor  Adhesion molecule  Transport/cargo protein  Unclassified  Unclassified  DNA binding protein  Cell cycle control protein  Ribosomal subunit  Transport/cargo protein  Translation regulatory protein  unknown  Enzyme: Deacetylase  GTPase  Serine/threonine kinase  Unclassified  Unclassified  Adapter molecule  Cell cycle control protein  GTPase  Enzyme: Sialyltransferase  Ubiquitin proteasome system protein  Unclassified  Unclassified  Serine/threonine kinase  Ion channel  Enzyme: Hydroxylase  Transcription factor  Calcium binding protein  Integral membrane protein  G protein coupled receptor  Transport/cargo protein  Translation regulatory protein  unknown  Enzyme: Sulphotransferase  Cytoskeletal associated protein  Intracellular ligand gated channel  Cell cycle control protein  Integral membrane protein  Chaperone  Unclassified  Transcription factor  Enzyme: Mutase  Enzyme: Phosphodiesterase  DNA repair protein  Transcription factor  Enzyme: Ligase  Transcription factor  G protein coupled receptor  G protein coupled receptor  Integral membrane protein  Enzyme: Prenyltransferase  Enzyme: Deaminase  Unclassified  Unclassified  Enzyme: Phosphotransferase  Transcription factor  DNA binding protein  Dual specificity kinase  Secreted polypeptide  RNA binding protein  Enzyme: Decarboxylase  RNA binding protein  Translation regulatory protein  Enzyme: Topoisomerase  DNA helicase  Unclassified  Membrane transport protein  Motor protein  Transcription factor  Aminopeptidase  Lipid Kinase  Unclassified  Transcription factor  Enzyme: Glycosidase  Transcription regulatory protein  Dual specificity phosphatase  RNA binding protein  Unclassified  Unclassified | Signaling  Cell cycle  unknown  Protein metabolism  signaling  unknown  unknown  unknown  Cell growth and/or maintenance  Cell growth and/or maintenance  nucleic acid metabolism  signaling  signaling  nucleic acid metabolism  signaling  nucleic acid metabolism  Protein metabolism  unknown  unknown  unknown  unknown  signaling  unknown  unknown  nucleic acid metabolism  Transport  unknown  unknown  unknown  Cell growth and/or maintenance  Cell growth and/or maintenance  Protein metabolism  Transport  Cell adhesion  Cell growth and/or maintenance  signaling  nucleic acid metabolism  signaling  Transport  signaling  nucleic acid metabolism  nucleic acid metabolism  unknown  nucleic acid metabolism  nucleic acid metabolism  Cell growth and/or maintenance  Apoptosis  Metabolism ; Energy pathways  Transport  signaling  unknown  nucleic acid metabolism  Transport  signaling  nucleic acid metabolism  Cell growth and/or maintenance  signaling  signaling  signaling  signaling  Protein metabolism  signaling  Cell growth and/or maintenance  signaling  nucleic acid metabolism  unknown  Metabolism ; Energy pathways  unknown  Metabolism ; Energy pathways  nucleic acid metabolism  unknown  Immune response  nucleic acid metabolism  unknown  signaling  signaling  signaling  nucleic acid metabolism  unknown  Cell growth and/or maintenance  Metabolism ; Energy pathways  unknown  signaling  Metabolism ; Energy pathways  nucleic acid metabolism  signaling  Cell proliferation  signaling  Gene silencing  signaling  Immune response  DNA repair  signaling  signaling  unknown  unknown  Metabolism ; Energy pathways  Transport  Cell growth and/or maintenance  signaling  signaling  signaling  signaling  Metabolism  Metabolism ; Energy pathways  nucleic acid metabolism  Cytoskeletal anchoring  Protein metabolism  nucleic acid metabolism  nucleic acid metabolism  signaling  nucleic acid metabolism  signaling  nucleic acid metabolism  signaling  signaling  nucleic acid metabolism  Cell growth and/or maintenance  Drug metabolism  Transport  unknown  unknown  signaling  nucleic acid metabolism  nucleic acid metabolism  Protein metabolism  nucleic acid metabolism  unknown  Transport  unknown  nucleic acid metabolism  signaling  Transport  unknown  Metabolism ; Energy pathways  Metabolism ; Energy pathways  nucleic acid metabolism  Immune response  Cell growth and/or maintenance  signaling  Metabolism ; Energy pathways  Transport  Metabolism ; Energy pathways  signaling  nucleic acid metabolism  unknown  nucleic acid metabolism  Ion transport  Cell adhesion  unknown  signaling  unknown  signaling  unknown  Regulation of cell cycle  signaling  unknown  nucleic acid metabolism  signaling  signaling  Transport  unknown  signaling  unknown  signaling  Protein metabolism  Transport  Protein metabolism  unknown  Metabolism ; Energy pathways  signaling  signaling  unknown  unknown  Transport  signaling  signaling  signaling  Protein metabolism  signaling  unknown  signaling  Transport  Metabolism ; Energy pathways  nucleic acid metabolism  signaling  signaling  signaling  Transport  Protein metabolism  unknown  Metabolism ; Energy pathways  Cell growth and/or maintenance  Transport  signaling  signaling  nucleic acid metabolism  unknown  nucleic acid metabolism  Metabolism ; Energy pathways  Metabolism ; Energy pathways  nucleic acid metabolism  nucleic acid metabolism  Metabolism ; Energy pathways  nucleic acid metabolism  signaling  signaling  unknown  Metabolism ; Energy pathways  Metabolism ; Energy pathways  unknown  signaling  Energy pathways  nucleic acid metabolism  nucleic acid metabolism  signaling  unknown  Protein metabolism  Metabolism ; Energy pathways  nucleic acid metabolism  Protein metabolism  nucleic acid metabolism  nucleic acid metabolism  Protein metabolism  Transport  Cell growth and/or maintenance  nucleic acid metabolism  Protein metabolism  signaling  Immune response  nucleic acid metabolism  Metabolism ; Energy pathways  nucleic acid metabolism  signaling  nucleic acid metabolism  Apoptosis  unknown | unknown  Cytoplasm  cytoplasm  Mitochondrion  unknown  unknown  unknown  unknown  Cytoplasm  Nucleus  Plasma membrane  Nucleus  Nucleus  Cytoplasm  Nucleus  Cytoplasm  unknown  unknown  unknown  unknown  unknown  unknown  unknown  unknown  Mitochondrion  unknown  unknown  unknown  Nucleus  Cytoplasm  unknown  unknown  Integral to membrane  Plasma membrane  Plasma membrane  Nucleus  Nucleus  Plasma membrane  Cytoplasm  Nucleus  Nucleus  unknown  Extracellular  Cytoplasm  Cell projection  Mitochondrion  Integral to membrane  Sarcoplasmic reticulum  Extracellular  Integral to membrane  Nucleus  Plasma membrane  Nucleus  Nucleus  Actin cytoskeletonl  Cytoplasm  unknown  Plasma membrane  Cytoplasm  unknown  Cytoplasm  Plasma membrane  Plasma membrane  Nucleus  Cytoplasm  Peroxisome  unknown  Cytoplasm  Nucleus  unknown  Cytoplasm  Nucleus  unknown  Plasma membrane  Cytoplasm  Cell surface  Nucleus  unknown  Extracellular  Cytoplasm  unknown  Extracellular  Cytoplasm  Nucleus  unknown  Cytoskeleton  Plasma membrane  Cytoplasm  unknown  unknown  Nucleus  Cytoplasmic vesicle  Plasma membrane  unknown  unknown  Mitochondrion  Plasma membrane  unknown  Nucleus  Plasma membrane  Cytosol  Extracellular  Mitochondrion  Cytoplasm  Nucleus  unknown  Extracellular  Nucleus  Nucleus  Cytoplasm  Cytoplasm  unknown  Nucleus  Plasma membrane  Cytoplasm  Nucleus  Extracellular  Endoplasmic reticulum  Golgi apparatus  Plasma membrane  unknown  Plasma membrane  Nucleus  Nucleus  Cytoplasm  Nucleus  unknown  Plasma membrane  unknown  Nucleus  Plasma membrane  Lysosome  unknown  Cytoplasm  unknown  Nucleus  unknown  Extracellular  Plasma membrane  Cytoplasm  Endoplasmic reticulum  Mitochondrion  Plasma membrane  Nucleus  unknown  Nucleus  Integral to membrane  unknown  unknown  unknown  unknown  Cytoplasm  Cytoplasm  Cytoplasm  Nucleus  unknown  unknown  Plasma membrane  Plasma membrane  Cytoplasm  unknown  Cytoplasm  Nucleus  Cytoplasm  Mitochondrion  Golgi apparatus  Mitochondrion  unknown  Endoplasmic reticulum  Plasma membrane  Cytoplasm  unknown  unknown  Plasma membrane  Nucleus  Integral to membrane  Golgi apparatus  Cytoplasm  Plasma membrane  Nucleus  Nucleus  Plasma membrane  Plasma membrane  Nucleus  Plasma membrane  Plasma membrane  Plasma membrane  Extracellular  Nucleus  unknown  Golgi apparatus  unknown  Plasma membrane  Nucleus  Integral to membrane  Nucleus  unknown  Nucleus  Cytoplasm  Cytoplasm  Nucleus  Nucleus  Cytoplasm  Nucleus  Plasma membrane  Plasma membrane  unknown  Cytoplasm  Extracellular  unknown  Plasma membrane  unknown  Nucleus  Nucleus  Nucleus  Extracellular  Nucleus  Cytoplasm  Nucleus  Mitochondrion  Mitochondrion  Nucleus  Nucleus  Integral to membrane  Cytoplasm  Nucleus  Plasma membrane  Plasma membrane  Nucleus  Nucleus  Plasma membrane  Nucleus  Nucleus  Nucleus  Cytoplasm and Nucleus  unknown | Liver  liver  liver  unknown  unknown  unknown  unknown  unknown  liver  liver  liver  unknown  liver and liver cancer  liver  liver  liver  liver  unknown  unknown  unknown  unknown  liver  unknown  unknown  liver  liver  unknown  liver  unknown  liver  liver  liver and liver cancer  unknown  unknown  liver and liver cancer  liver  liver  liver  liver  liver  liver and liver cancer  unknown  unknown  liver  liver  liver  liver  liver  liver  unknown  unknown  unknown  unknown  liver  unknown  liver  unknown  liver  liver  liver and liver cancer  liver  liver  liver  unknown  unknown  liver  liver  unknown  liver and liver cancer  unknown  liver  unknown  liver  unknown  unknown  liver  liver and liver cancer  liver  unknown  unknown  liver  unknown  unknown  liver  liver  unknown  liver  liver  liver and liver cancer  liver  liver cancer  liver cancer  unknown  liver  unknown  liver  unknown  unknown  liver  liver  unknown  liver and liver cancer  unknown  liver  liver and liver cancer  liver  liver  liver  liver  liver  liver  liver and liver cancer  unknown  liver  liver  unknown  liver  liver  liver  unknown  liver  unknown  liver  liver  unknown  unknown  liver  unknown  liver  unknown  liver  unknown  liver  liver  liver  liver  unknown  unknown  liver  liver and liver cancer  liver  unknown  liver  unknown  liver and liver cancer  unknown  unknown  liver  liver  unknown  unknown  unknown  liver  liver  unknown  unknown  unknown  liver  liver  liver  liver  liver  unknown  liver  liver  unknown  liver  liver  unknown  liver  liver  liver cancer  liver  unknown  unknown  liver  liver  liver  liver  liver  liver  unknown  unknown  liver  liver  liver  liver  liver  liver  liver and liver cancer  unknown  unknown  liver  unknown  unknown  unknown  liver  liver  unknown  liver and liver cancer  liver  liver  liver and liver cancer  unknown  liver  unknown  unknown  unknown  liver cancer  liver  unknown  liver  liver  liver  unknown  unknown  unknown  liver  liver  liver  unknown  liver  liver  unknown  liver  liver  liver  unknown  liver  liver  unknown  unknown  unknown  unknown  liver  unknown  unknown |
