# Supplementary material for: Identification of tyrosine-phosphorylated proteins associated with metastasis and functional analysis of FER in human hepatocellular carcinoma cells
Source: BMC Cancer. 2009 Oct 16;9:366. doi: 10.1186/1471-2407-9-366 (PMC2770568; doi:10.1186/1471-2407-9-366)
Supplement: Additional file 3 — Supplemental Table S5. Table S5a: Pathway clustering for node proteins in PPI network of MHCC97H cell. Table S5b: Pathway clustering for node proteins in PPI network of Hep3B cell. Legend: The two tables summarize detailed information of pathway clustering results generated from BinGO program, which was run to explore the functional regulation of node proteins in PPI network of MHCC97H and Hep3B cell. [file 1471-2407-9-366-S3.DOC]

| GO-pathway name  **Table 5a Pathway clustering for node proteins in PPI network of MHCC97H cell** | corr p-value | Protein number | Cluster freq. | Protein |
| --- | --- | --- | --- | --- |
| **Sigal transduction**  **Cell surface receptor linked signal transduction**  **Intracellular signaling cascade**  **lipopolysaccharide-mediated signaling pathway**  **integrin-mediated signaling pathway**  **glutamate signaling pathway**  **cytokine and chemokine mediated signaling pathway**  **Wnt receptor signaling pathway**  **enzyme linked receptor protein signaling pathway**  **SMAD protein complex assembly**  **transforming growth factor beta receptor signaling pathway**  **transmembrane receptor protein tyrosine kinase signaling pathway**  **epidermal growth factor receptor signaling pathway**  **signal complex assembly**  **insulin-like growth factor receptor signaling pathway**  **insulin receptor signaling pathway**  **phosphoinositide-mediated signaling**  **small GTPase mediated signal transduction**  **Ras protein signal transduction**  **intracellular receptor-mediated signaling pathway**  **steroid hormone receptor signaling pathway**  **androgen receptor signaling pathway**  **protein kinase cascade**  **phosphoinositide 3-kinase cascade**  **JAK-STAT cascade**  **tyrosine phosphorylation of STAT protein**  **peptidyl-tyrosine phosphorylation**  **protein amino acid phosphorylation**  **peptidyl-serine phosphorylation**  **protein amino acid autophosphorylation**  **MAPKKK cascade**  **inactivation of MAPK activity**  **positive regulation of MAP kinase activity**  **I-kappaB kinase/NF-kappaB cascade**  **activation of NF-kappaB-inducing kinase activity**  **positive regulation of phosphoinositide 3-kinase activity**  **regulation of phosphoinositide 3-kinase activity**  **protein amino acid autophosphorylation** | 4.20E-25  5.89E-10  1.63E-21  2.82E-02  1.77E-02  7.59E-05  1.11E-04  2.63E-03  7.52E-15  2.82E-02  7.74E-03  3.29E-10  9.39E-05  2.51E-02  7.77E-03  2.14E-06  2.12E-02  3.02E-02  2.07E-04  5.19E-03  2.42E-03  7.02E-03  2.19E-12  2.33E-04  2.33E-05  4.13E-02  1.35E-02  1.60E-15  3.68E-02  5.61E-05  2.10E-04  2.15E-02  2.02E-02  2.65E-02  4.16E-03  1.72E-02  1.72E-02  5.61E-05 | 230  114  115  2  7  6  9  12  42  2  6  29  7  3  3  9  10  19  13  8  8  6  40  4  8  2  4  69  3  10  16  4  8  6  4  2  2  10 | 43.7%  21.7%  21.9%  0.3%  1.3%  1.1%  1.7%  2.3%  8%  0.3%  1.1%  5.5%  1.3%  0.6%  0.6%  1.7%  1.9%  3.6%  2.5%  1.5%  1.5%  1.1%  7.6%  1.1%  1.5%  0.3%  0.8%  13.1%  0.6%  1.9%  3%  0.8%  1.5%  1.1%  0.8%  0.3%  0.3%  1.9% | HNRPK|HRAS|IL6ST|STAT5A|STAT5B|STOML3|MAP3K7|APP|GRIN2B|GRIN2D|ILK|CHRNA7|GUCA2A|CCNA2|STAG1|SYK|PTPRJ|PLD2|BSG|PLD1|PTPRF|STRN4|CD40|MARK4|HTATIP|MAPK1|CCR5|MAPK3|MAPK9|RYR2|PDGFRB|MAPK8|EIF2AK2|ERBB2|NFKBIA|ADRBK1|INS|RAC1|DVL2|TGFBR1|PHB|CREBBP|SMAD4|S100A11|SMAD3|SKI|SMAD2|ATR|SMAD1|GUCY2C|HOMER1|ATM|PORCN|PTPN11|NOTCH3|NOTCH1|LAMA3|RGS2|ARF1|SFRP1|NUP62|SFRP2|RGS4|ZBTB33|PDIA3|NFKB1|PTEN|CCNE1|EDNRB|PDPK1|BAG1|IL1RAP|TOP2A|CEACAM1|BRD8|AR|ARHGEF7|SF1|RASIP1|NDUFA13|IL6R|TNFRSF10A|TRAP1|CCND1|TNFRSF10C|TNFRSF10B|TNFRSF10D|GTF2I|NCK1|PSEN2|THOC1|YWHAZ|PARD3|MLLT7|CCR1|PML|FPR1|IL7R|SOS1|PER2|APBA3|PER1|HBP1|ACTL6A|RASA2|NLK|YWHAB|GRIA4|STAT1|YWHAE|ITPR1|STAT3|FZD6|RPS6KA5|RPS6KA1|CSNK1D|ERBB2IP|CSNK1E|FYN|GSK3B|DGKZ|APBB3|APBB2|APBB1|DNM2|F2RL2|F2RL3|GRIK2|GRIK3|GNA12|RCVRN|PRKCB1|EIF4EBP1|PRKAR2A|WNT4|EDG1|PRKACA|EGFR|RET|FRAP1|RELA|FADD|IRS1|AHR|HIF1A|EP300|STMN1|DST|SRI|TRAF2|FGFR1|CAV1|FGFR4|FGFR3|GRB2|ITGB4|TRRAP|SRF|ITGB1|SRC|ATF2|VDR|NPM1|PTPN6|GABRA1|GABRA4|MET|GRIN1|BMX|NR4A2|SHANK2|BRCA1|TNFSF10|PTPN1|SMC1A|ABL1|GRB7|PDZD3|NXPH3|NBN|LEPR|RHOQ|PIP5K1A|FER|CDC42|CASP3|CXCR4|RANBP3|CSF2RB|SHC1|ARHGAP8|SHC3|PRKCA|KHDRBS1|HSP90AA1|ESR1|PRKCG|RB1|PRKCE|PRKCD|CDK5|IFNAR1|IFNAR2|NCOA1|LCK|NCOA6|TXNRD1|TSHR|PRKCZ|CTNND2|CTNND1|NR3C1|IGF1R|APPBP1|PIK3R3|PIK3R1|CEBPA|IL2RB|IL2RA|FLT1|NF1|ANXA1|RAF1|BAD|GMFB|NCSTN|JAK1|JAK2|CRK|FEZ2  F2RL2|F2RL3|HRAS|GRIK2|IL6ST|GRIK3|STAT5A|GNA12|STAT5B|MAP3K7|EIF4EBP1|APP|WNT4|GRIN2B|ILK|EDG1|SYK|EGFR|PTPRJ|BSG|PTPRF|RELA|IRS1|MARK4|MAPK1|CCR5|PDGFRB|DST|FGFR1|FGFR4|FGFR3|GRB2|ERBB2|ITGB4|NFKBIA|ADRBK1|ITGB1|SRC|INS|DVL2|PTPN6|GABRA1|GABRA4|TGFBR1|MET|GRIN1|SMAD4|SMAD3|SKI|SMAD1|GUCY2C|HOMER1|PORCN|NOTCH3|NOTCH1|LAMA3|RGS2|SFRP1|NUP62|SFRP2|PTPN1|GRB7|PDZD3|NXPH3|ZBTB33|LEPR|PTEN|EDNRB|PDPK1|BAG1|CXCR4|CSF2RB|SHC1|SHC3|CEACAM1|BRD8|KHDRBS1|IL6R|CDK5|IFNAR1|IFNAR2|TRAP1|TNFRSF10B|NCK1|PSEN2|TSHR|PARD3|MLLT7|CCR1|FPR1|CTNND1|IL7R|IGF1R|HBP1|PIK3R3|PIK3R1|CEBPA|IL2RB|IL2RA|FLT1|ANXA1|RAF1|GRIA4|STAT3|FZD6|RPS6KA5|NCSTN|CSNK1D|ERBB2IP|FYN|GSK3B|DGKZ|JAK1|JAK2  F2RL3|HRAS|STAT5A|STAT5B|PRKCB1|MAP3K7|PRKAR2A|EDG1|CHRNA7|PRKACA|CCNA2|SYK|STAG1|EGFR|PLD2|PLD1|FADD|IRS1|HTATIP|MAPK1|MAPK3|MAPK9|MAPK8|STMN1|EIF2AK2|TRAF2|FGFR1|CAV1|FGFR3|GRB2|ERBB2|NFKBIA|SRC|INS|RAC1|DVL2|PTPN6|MET|BMX|SMAD3|SMAD2|ATR|SMAD1|GUCY2C|HOMER1|SHANK2|BRCA1|TNFSF10|ARF1|NUP62|RGS4|SMC1A|ABL1|PDZD3|NBN|ZBTB33|RHOQ|FER|CDC42|CCNE1|EDNRB|CASP3|PDPK1|CXCR4|RANBP3|SHC1|SHC3|TOP2A|PRKCA|ARHGEF7|ESR1|NDUFA13|PRKCG|RB1|PRKCE|PRKCD|IFNAR1|TNFRSF10A|IFNAR2|CCND1|NCOA1|TNFRSF10B|LCK|NCOA6|PSEN2|TSHR|PRKCZ|PARD3|CCR1|PML|FPR1|IGF1R|SOS1|APBA3|APPBP1|PIK3R1|RASA2|NLK|NF1|YWHAB|RAF1|BAD|STAT1|YWHAE|STAT3|RPS6KA5|RPS6KA1|FYN|GSK3B|DGKZ|JAK1|JAK2|APBB3|APBB2|CRK  MAPK1|NFKBIA  ERBB2IP|ILK|ITGB4|ITGB1|DST|CEACAM1|SYK  GRIN2B|GRIK2|GRIK3|GRIN1|GRIA4|HOMER1  CEBPA|IL2RB|CCR1|STAT5A|RELA|STAT5B|JAK1|CSF2RB|STAT3  DVL2|WNT4|ZBTB33|SFRP1|CSNK1D|SFRP2|GSK3B|CTNND1|HBP1|MARK4|PORCN|FZD6  FGFR1|FGFR4|MLLT7|FGFR3|GRB2|ERBB2|PTEN|SRC|MAP3K7|IGF1R|PDPK1|EIF4EBP1|SHC1|PIK3R3|SHC3|PIK3R1|SYK|EGFR|PTPRJ|FLT1|PTPRF|TGFBR1|MET|SMAD4|RAF1|SMAD3|SKI|SMAD1|GUCY2C|IRS1|CDK5|RPS6KA5|TRAP1|ERBB2IP|RGS2|NCK1|JAK1|PDGFRB|JAK2|PTPN1|GRB7|PDZD3  SMAD4|SMAD3  MAP3K7|TRAP1|TGFBR1|SMAD4|SMAD3|SMAD1  FGFR1|MLLT7|FGFR4|FGFR3|GRB2|ERBB2|PTEN|SRC|IGF1R|EIF4EBP1|PDPK1|SHC1|PIK3R3|SHC3|PIK3R1|EGFR|PTPRJ|FLT1|MET|RAF1|CDK5|IRS1|RPS6KA5|RGS2|ERBB2IP|NCK1|PDGFRB|PTPN1|GRB7  EGFR|RPS6KA5|ERBB2IP|GRB2|SHC1|SHC3|GRB7  NCK1|CDK5|SRC  IGF1R|IRS1|PIK3R1  IGF1R|MLLT7|PDPK1|EIF4EBP1|GRB2|PTPN1|PIK3R3|IRS1|PIK3R1  EGFR|F2RL3|EDNRB|IGF1R|PARD3|ERBB2|EDG1|DGKZ|HOMER1|TOP2A  PLD2|HRAS|PLD1|GRB2|NF1|YWHAB|RHOQ|RAF1|SRC|MAPK1|CDC42|ARF1|SOS1|MAPK3|RAC1|LCK|RANBP3|SHC1|SHC3  HRAS|PLD1|GRB2|NF1|YWHAB|RAF1|SRC|MAPK1|SOS1|MAPK3|LCK|SHC1|SHC3  CCNE1|NCOA1|NCOA6|ESR1|RB1|HTATIP|BRCA1|STAG1  CCNE1|NCOA1|NCOA6|ESR1|RB1|HTATIP|BRCA1|STAG1  CCNE1|NCOA1|RB1|HTATIP|BRCA1|STAG1  FGFR1|TRAF2|CAV1|FGFR3|ERBB2|STAT5A|STAT5B|FPR1|NFKBIA|SRC|MAP3K7|IGF1R|CXCR4|INS|SHC1|PRKACA|CHRNA7|SYK|PRKCA|PLD1|NLK|MET|NF1|SMAD1|STAT1|IRS1|STAT3|IFNAR1|RPS6KA5|TNFRSF10A|IFNAR2|MAPK1|TNFRSF10B|RPS6KA1|FYN|RGS4|MAPK9|JAK1|MAPK8|JAK2  IGF1R|INS|ERBB2|NF1  IFNAR2|FGFR3|STAT5A|STAT5B|JAK2|STAT1|STAT3|IFNAR1  JAK2|STAT1  JAK1|JAK2|STAT1|ABL1  MYOD1|FER|PRKCB1|FES|MAP3K7|PDPK1|CXCR4|ILK|EEF2K|SHC1|PRKACA|CHRNA7|SYK|EGFR|RET|CTBP1|CDC2|TRPM7|CDK9|PRKCG|PRKCE|CDK4|PRKCD|MARK4|CDK5|CDK2|MAPK1|MAPK3|LCK|MAPK9|PDGFRB|MAPK8|EIF2AK2|FRK|PRKCZ|CAV2|FGFR1|FGFR4|FGFR3|ERBB2|FPR1|PRKDC|ADRBK1|SRC|IGF1R|PRKRA|TAF1|FLT1|TGFBR1|HCK|NLK|MET|BMX|RAF1|STAT1|GUCY2C|GMFB|RPS6KA5|CDKN1A|RPS6KA1|CSNK1D|FYN|GSK3A|CSNK1E|GSK3B|JAK1|JAK2|JAK3|ABL1  GSK3B|PRKDC|ADRBK1  CAV2|IGF1R|TAF1|NLK|ERBB2|MET|PDGFRB|FER|EIF2AK2|SYK  PRKCA|FGFR1|CAV1|FGFR3|MET|FPR1|SMAD1|MAPK1|CXCR4|RGS4|MAPK9|SHC1|MAPK8|CHRNA7|PRKACA|SYK  PRKCA|CAV1|RGS4|PRKACA  EGFR|CXCR4|ERBB2|MET|FPR1|SHC1|CHRNA7|SYK  TNFRSF10A|MAP3K7|TRAF2|TNFRSF10B|NFKBIA|STAT1    TNFRSF10A|MAP3K7|TRAF2|TNFRSF10B  CDC42|IRS1  CDC42|IRS1  CAV2|IGF1R|TAF1|NLK|ERBB2|MET|PDGFRB|FER|EIF2AK2|SYK |

**Table 5b Pathway clustering for node proteins in PPI network of Hep3B cell**

| GO-pathway name | Corr p-value | Protein number | Cluster freq. | Proteins |
| --- | --- | --- | --- | --- |
| **Signal transduction**  **metabotropic glutamate receptor signaling pathway**  **glutamate signaling pathway**  **enzyme linked receptor protein signaling pathway**  **transmembrane receptor protein tyrosine kinase signaling pathway**  **signal complex assembly**  **insulin receptor signaling pathway**  **insulin-like growth factor receptor signaling pathway**  **intracellular signaling cascade**  **small GTPase mediated signal transduction**  **Ras protein signal transduction**  **stress-activated protein kinase signaling pathway**  **JNK cascade**  **protein kinase cascade**  **MAPKKK cascade**  **protein kinase B signaling cascade**  **protein amino acid dephosphorylation** | 8.23E-10  2.12E-02  2.34E-02  1.88E-04  9.00E-05  4.91E-02  2.38E-06  2.59E-02  9.19E-14  5.46E-05  1.91E-04  3.78E-02  3.21E-02  8.97E-11  2.38E-03  6.05E-03  2.40E-02 | 97  2  3  15  13  2  7  2  60  17  9  4  4  25  9  2  7 | 42.5%  0.9%  0.9%  6.6%  5.7%  0.9%  3.1%  0.9%  26.3%  7.5%  3.9%  1.8%  1.8%  11.0%  3.9%  0.9%  3.1% | STAT5A|GNA12|RAB1B|RPS6KB1|PRKCB1|EIF4EBP1|APOA1|EIF4EBP2|HOMER3|GRIN2D|ILK|FAS|MX1|EGFR|PLD2|PLD1|RELA|IRS1|DDIT3|MAPK1|EP300|MAPK3|MAPK9|MAPK8|CAV1|CCL3|BLM|FOXO1A|ELK1|SRF|SRC|RAC2|RAC1|MDFI|MAP2K1|CREBBP|GRIN1|S100A11|SMAD4|BRIP1|HOMER1|BRCA1|P2RX7|ADRA1B|PTPN1|NFKB1|PTEN|AKT1|PDPK1|RHOA|TGFA|CAP1|AKT3|PRKCA|SRPK2|ARL1|AR|HSP90AA1|LYN|RALBP1|RAB4A|PRKCI|ESR1|PKN2|PKN1|PRKCG|RB1|PRKCE|HMGA1|PRKCD|SRPK1|CARD11|ADRB1|PRKCZ|RAB3D|MKNK2|MKNK1|NR3C1|STAT6|PTK2B|PKD2|TGM2|PTN|CREB1|ITPR3|FZD4|RALGDS|ITPR2|RPS6KA5|DUSP4|RPS6KA3|YWHAH|RPS6KA4|RPS6KA1|GRIA1|MAPK14|APAF1  HOMER3|HOMER1  HOMER3|GRIN1|HOMER1  EGFR|FOXO1A|SMAD4|HMGA1|PTEN|IRS1|SRC|RPS6KA5|AKT1|PDPK1|EIF4EBP1|EIF4EBP2|PTK2B|PTN|PTPN1  EGFR|FOXO1A|PTEN|IRS1|HMGA1|SRC|RPS6KA5|AKT1|PDPK1|EIF4EBP1|EIF4EBP2|PTK2B|PTPN1  PTK2B|SRC  AKT1|EIF4EBP1|PDPK1|EIF4EBP2|FOXO1A|PTPN1|IRS1  AKT1|IRS1  STAT5A|RAB1B|PRKCB1|AKT1|PDPK1|APOA1|EIF4EBP2|RHOA|TGFA|FAS|EGFR|PRKCA|SRPK2|PLD2|ARL1|PLD1|LYN|RALBP1|RAB4A|ESR1|PRKCI|PKN1|PRKCG|RB1|PRKCE|PRKCD|IRS1|SRPK1|DDIT3|MAPK1|ADRB1|MAPK3|MAPK9|MAPK8|PRKCZ|CAV1|RAB3D|BLM|MKNK2|MKNK1|SRC|RAC2|RAC1|TGM2|PKD2|MDFI|MAP2K1|BRIP1|HOMER1|RALGDS|BRCA1|RPS6KA5|DUSP4|RPS6KA3|RPS6KA4|YWHAH|RPS6KA1|MAPK14|ADRA1B|APAF1  ARL1|PLD2|PLD1|MAP2K1|RAB3D|RALBP1|RAB4A|RAB1B|RALGDS|SRC|MAPK1|APOA1|RAC2|MAPK14|MAPK3|RAC1|RHOA  MAPK1|PLD1|APOA1|MAP2K1|MAPK14|MAPK3|RHOA|RALGDS|SRC  MDFI|PKN1|MAPK9|MAPK8  MDFI|PKN1|MAPK9|MAPK8  CAV1|STAT5A|MKNK2|MKNK1|SRC|AKT1|PKD2|TGFA|MDFI|PRKCA|SRPK2|PLD1|PKN1|IRS1|SRPK1|RPS6KA5|MAPK1|DUSP4|RPS6KA3|RPS6KA4|RPS6KA1|MAPK14|ADRA1B|MAPK9|MAPK8  PRKCA|MDFI|DUSP4|MAPK1|CAV1|PKN1|TGFA|MAPK9|MAPK8  AKT1|IRS1  PTPRB|DUSP4|SSH1|SSH3|SSH2|PTPN1|PTEN |
